# Staggered immunization with mRNA vaccines encoding SARS-CoV-2 polymerase or spike antigens broadens the T cell epitope repertoire

**DOI:** 10.1073/pnas.2406332121

**Published:** 2024-11-26

**Authors:** Evan R. Abt, Alex K. Lam, Miyako Noguchi, Khalid Rashid, Jami McLaughlin, Pu-Lin Teng, Wendy Tran, Donghui Cheng, Pavlo A. Nesterenko, Zhiyuan Mao, Amanda L. Creech, Giselle Burton Sojo, Arjit Vijey Jeyachandran, Ying K. Tam, Jill E. Henley, Lucio Comai, Norbert Pardi, Vaithilingaraja Arumugaswami, Owen N. Witte, Caius G. Radu, Ting-Ting Wu

**Affiliations:** ^a^Department of Molecular and Medical Pharmacology, University of California Los Angeles, Los Angeles, CA 90095; ^b^Department of Microbiology, Immunology, and Molecular Genetics, University of California Los Angeles, Los Angeles, CA 90095; ^c^Acuitas Therapeutics, Vancouver, BC V6T 1Z3, Canada; ^d^Department of Molecular Microbiology and Immunology, The Hastings and Wright Laboratories, Keck School of Medicine, University of Southern California, Los Angeles, CA 90089; ^e^Department of Microbiology, Perelman School of Medicine, University of Pennsylvania, Philadelphia, PA 19104; ^f^Jonsson Comprehensive Cancer Center, University of California Los Angeles, Los Angeles, CA 90095; ^g^Molecular Biology Institute, University of California Los Angeles, Los Angeles, CA 90095; ^h^Eli and Edythe Broad Center of Regenerative Medicine and Stem Cell Research, University of California Los Angeles, Los Angeles, CA 90095; ^i^Parker Institute for Cancer Immunotherapy, University of California Los Angeles, Los Angeles, CA 90095; ^j^AIDS Institute, David Geffen School of Medicine, University of California Los Angeles, Los Angeles, CA 90095

**Keywords:** mRNA vaccines, T cell receptor specificity, interferon signaling, SARS-CoV-2, anti-pathogen T cell response

## Abstract

Combining a T cell-targeting mRNA vaccine encoding the conserved SARS-CoV-2 RNA-dependent RNA polymerase, RdRp, with a Spike-encoding mRNA vaccine may offer an additional pathway toward COVID-19 protection. Here, we show that a nucleoside-modified RdRp mRNA vaccine raises robust and durable CD8+ T cell responses in mice. Immunization drives a CD8+ T cell response enriched toward a specific RdRp epitope. Unexpectedly, coadministration of mRNA vaccines encoding RdRp or the Spike Receptor Binding Domain (RBD) dampens RBD-specific immune responses. Contralateral administration reduces the suppression of RBD-specific T cell responses while type I interferon signaling blockade restores RBD-specific antibodies. A staggered immunization strategy maintains both RBD vaccine-mediated antibody and T cell responses as well as protection against lethal SARS-CoV-2 challenge in human ACE2 transgenic mice. In HLA-A2.1 transgenic mice, the RdRp vaccine elicits CD8+ T cell responses against HLA-A*02:01-restricted epitopes recognized by human donor T cells. These results highlight RdRp as a candidate antigen for COVID-19 vaccines. The findings also offer insights into crafting effective multivalent mRNA vaccines to broaden CD8+ T cell responses against SARS-CoV-2 and potentially other viruses with pandemic potential.

## Significance

Immunization to expand SARS-CoV-2-specific immune responses against non-Spike epitopes may represent a path toward preventing severe COVID-19 in the face of viral evolution. We generated a T cell-targeting mRNA vaccine encoding the infrequently altered SARS-CoV-2 RNA polymerase RdRp. In mice, this vaccine stimulates robust and durable CD8+ T cell responses. Unexpectedly, when this vaccine is coadministered with a SARS-CoV-2 Spike RBD-encoding vaccine, Spike-specific antibody and T cell responses are dampened. However, a staggered immunization strategy preserves Spike-specific immune responses. This research provides insights relevant to the development of mRNA vaccine-based immunization strategies for future SARS-CoV-2 variants, other infectious agents, or cancer.

Current SARS-CoV-2 mRNA vaccines effectively raise neutralizing antibody and T cell responses specific for Spike protein epitopes (1–4). Broadening the SARS-CoV-2-specific immune repertoire through coadministration with vaccines encoding Spike and conserved non-Spike epitopes may provide additional protection against emerging variants.

While neutralizing antibodies provide a first line of defense by preventing viruses from infecting cells, CD8+ T cells can eliminate infected cells by recognizing viral epitopes presented by major histocompatibility complex (MHC) class I molecules on the cell surface. Evidence for a protective role of CD8+ T cells against SARS-CoV-2 is provided by preclinical models where infection drives antibody and T cell responses, and CD8+ cell depletion diminishes protection upon rechallenge (5). In a cohort of human cancer patients with impaired humoral immunity, CD8+ T cell responses were associated with improved COVID-19 recovery (6). mRNA vaccines for SARS-CoV-2 effectively elicit Spike-specific T cell and antibody responses in humans (7–9). The benefit of broadening the SARS-CoV-2 specific T cell response to target non-Spike epitopes through immunization is under investigation in a clinical trial testing mRNA vaccine BNT162b4, which encodes immunogenic variant-conserved segments of Nucleocapsid, Membrane, and ORF1ab proteins (NCT05541861) (10).

We have previously identified the ORF1b-encoded intracellular protein RNA-dependent RNA polymerase (RdRp), also known as Non-Structural Protein 12 (NSP12), as a priority antigen for T cell-targeting SARS-CoV-2 vaccines (11). RdRp is more highly conserved across beta-coronaviruses than Spike, Nucleocapsid, or Membrane proteins, indicating a key functional role that restrains its capacity for alteration (11). The detection of RdRp-reactive T cells in SARS-CoV-2 convalescent patients suggests the existence of SARS-CoV-2 RdRp-derived epitopes that prime T cell responses (12, 13). Human T cells expressing RdRp-specific T cell receptors (TCRs) eradicate RdRp-expressing targets and cross-react with homologous epitopes derived from multiple human coronaviruses (11). Raising RdRp-specific responses through immunization may contribute to protection against circulating and future coronavirus strains.

Nucleoside-modified mRNA encapsulated in lipid nanoparticles containing ionizable lipids (mRNA-iLNP) has emerged as a powerful viral vaccine platform to elicit cellular and antibody-mediated immunity (14–16). N1-methyl-pseudouridine (m1Ψ)-modified mRNA delivered by iLNP evades detection by intracellular RNA sensors to enable high levels of encoded protein production and the presentation of immunogen-derived peptides by MHC molecules in antigen-presenting cells (APCs) (17). mRNA-iLNP also provide adjuvant activity by inducing cytokines such as type I interferon (IFN) and interleukin-1 (IL-1) (18–20). By providing large amounts of antigen alongside cytokine signals, mRNA-iLNP prime strong and persistent antibody and T cell responses (21, 22).

To enhance T cell-mediated SARS-CoV-2 protection by broadening the virus-specific T Cell repertoire, we developed an mRNA-iLNP vaccine candidate encoding the conserved and infrequently altered RNA polymerase RdRp. The RdRp mRNA vaccine stimulates robust and durable RdRp-specific CD8+ T cell responses in C57BL/6 mice. When administered to HLA-A2.1 transgenic mice, the RdRp vaccine elicits T cells against HLA-A*02:01-restricted RdRp epitopes, previously shown to be recognized by human donor T cells (11). Cloning and functional validation of RdRp-specific murine TCRs isolated by single-cell sequencing revealed an immunodominant epitope encoded by the vaccine. Unexpectedly, coadministration of the RdRp vaccine with a SARS-CoV-2 Spike Receptor Binding Domain (RBD)-encoding mRNA vaccine resulted in suppressed RBD-specific T cell and antibody responses. A staggered immunization approach preserved RBD-specific immune responses and RBD vaccine-mediated protection against a lethal SARS-CoV-2 challenge in human ACE2 transgenic mice.

## Results

### A mRNA Vaccine Encoding the SARS-CoV-2 Polymerase RdRp Induces Antigen-Specific CD8+ T Cell Responses in Murine Models.

Conserved and infrequently altered viral epitopes are priority candidates for inclusion in mRNA vaccines for SARS-CoV-2. RdRp is conserved across beta-coronaviruses, and only three nonsynonymous RdRp mutations are found in SARS-CoV-2 variants that have replaced the ancestral strain (SI Appendix, Fig. S1A) (11). In contrast, over 40 nonsynonymous Spike mutations have been identified across SARS-CoV-2 variants (3). To investigate the potential T cell immunogenicity of a RdRp-targeting mRNA vaccine, we generated an m1Ψ-modified mRNA construct encoding a portion of the SARS-CoV-2 RdRp protein (SI Appendix, Fig. S1B). This mRNA encodes the N-terminal 611 amino acids of the Wuhan SARS-CoV-2 strain RdRp protein fused with an N-terminal MHC class I signal peptide and a C-terminal MHC I trafficking domain to enhance epitope presentation to T cells (23). The nucleoside-modified RdRp mRNA construct was generated by in vitro transcription with silica-membrane purification (24). The mRNA was encapsulated in iLNP for vaccine preparation (25). Antigen expression was confirmed by transfection of HEK293T cells (SI Appendix, Fig. S1C).

**Fig. 1.**
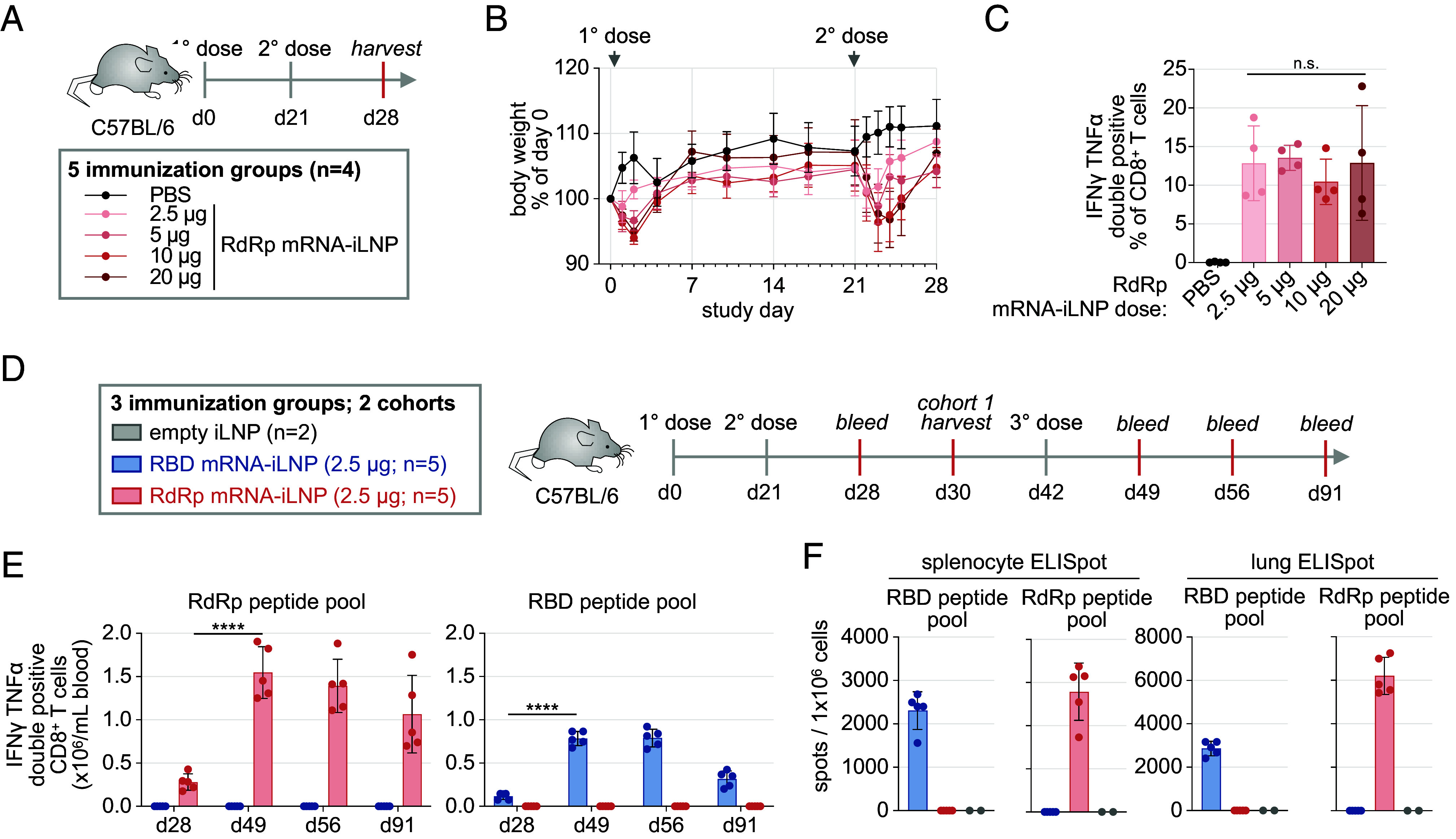
RdRp mRNA vaccine immunization elicits an antigen-specific CD8+ T cell response in mice. (*A*) Experimental design to test the T cell immunogenicity of a RdRp mRNA-iLNP vaccine in C57BL/6 mice (n = 4/group; i.m.). (*B*) Body weight change following immunization. (*C*) Intracellular cytokine staining (ICS) analysis of day 28 splenocytes restimulated with a RdRp peptide pool (mean ± SD; n = 4; one way ANOVA). (*D*) Experimental design to profile RdRp-specific and RBD-specific T cell responses in mice receiving two or three doses of the RBD mRNA-iLNP, the RdRp mRNA-iLNP or an empty iLNP control. (*E*) ICS analysis of CD3+/ CD8+ peripheral blood mononuclear cells (PBMC) from immunized mice restimulated with RdRp or RBD peptide pools (mean ±SD; one way ANOVA). (*F*) ELISpot analysis of day 30 splenocytes and lung cells pulsed with RdRp or RBD peptide pools. n.s.: not significant; **** *P* < 0.0001.

The T cell immunogenicity of the RdRp mRNA vaccine was defined in cohorts of C57BL/6 mice immunized via intramuscular injection with vaccine doses ranging from 2.5 µg to 20 µg (Fig. 1*A*). 21 d after primary immunization, a boost dose was administered. A transient decrease in the body weight was observed following immunizations, and such decreases were greater when higher doses were used (Fig. 1*B*). Published research has demonstrated that the ionizable lipid in iLNP drives an inflammatory response (26) Consequently, increasing dosages of the iLNP are expected to result in heightened inflammation, which is likely responsible for the observed transient weight loss.

To evaluate T cell responses, mice were sacrificed for splenocyte IFNγ and TNFα ICS and ELISpot analyses on day 7 following boost immunization. Splenocytes were restimulated ex vivo with a pool of sliding window overlapping 15mer RdRp peptides. A substantial fraction (10%) of the splenic CD8+ T cell population produced IFNγ and TNFα following restimulation (Fig. 1*C*). IFNγ ELISpot analysis of splenocytes restimulated with the RdRp peptide pool confirmed the presence of RdRp-specific T cells (SI Appendix, Fig. S1D). There was no significant difference in the proportion of RdRp-specific T cells as measured by IFNγ/TNFα ICS or IFNγ spot counts in mice receiving mRNA vaccine doses of or greater than 2.5 µg (Fig. 1*C* and SI Appendix, Fig. S1D). In contrast, no reactivity to 15mer RdRp peptide pool restimulation was observed by IFNγ and TNFα ICS at any tested time point within the CD4+ T cell population. In a separate study, RdRp-specific CD4+ T cells after two immunizations were detected by the activation-induced marker (AIM) assay (27). When CD69 and 4-1BB are used in different combinations for AIM in CD4+ T cells, the percentages of CD69+/4-1BB+ double-positive CD4+ T cells, as well as CD69+ CD4+ T cells are significantly higher than the control group immunized with an mRNA vaccine encoding hemagglutinin of influenza A virus (SI Appendix, Fig. S1E). These data indicate that the RdRp-encoding mRNA vaccine elicits robust and polyfunctional antigen-specific CD8+ T cell responses in C57BL/6 mice and that a 2.5 µg dose administered via intramuscular injection is sufficient for maximal T cell responses with no body weight alterations.

### Epitope Specificity of RdRp mRNA Vaccine-Elicited T Cells in HLA-A2.1 Transgenic Mice.

Specific HLA-A*02:01-restricted SARS-CoV-2 RdRp peptide epitopes activate human donor T cells collected prior to the COVID-19 pandemic (11). HLA-A2.1 transgenic mice were utilized to determine whether the RdRp mRNA vaccine raises T cells that recognize the same peptides (SI Appendix, Fig. S1F). HLA-A2.1 transgenic mice express a chimera of the extracellular domain of the human HLA-A2.1 molecule and the transmembrane and intracellular domain of the murine H-2D^b^ MHC class I molecule (28). This strain models T cell responses to epitopes presented on HLA-A*02:01 and provides a more robust T Cell repertoire than unmodified HLA-A2.1 due to improved positive selection of murine T cells. Transgenic HLA-A2.1 mice received 2.5 µg primary and boost doses of the RdRp mRNA vaccine and were sacrificed 11 d after the boost immunization. IFNγ ELISpot analysis of splenocytes following RdRp peptide pool restimulation indicated that immunization elicits robust RdRp-specific T cell responses in this strain (SI Appendix, Fig. S1F). These vaccine-elicited T cells were reactive to two of the three HLA-A*02:01-restricted RdRp epitopes defined in our previous work to be recognized by human donor T cells (11). This result expands on the previous finding by demonstrating that T cell responses specific for immunogenic HLA-A*02:01-restricted RdRp epitopes can be elicited by an mRNA vaccine.

### CD8+ T Cell Persistence, Phenotype, and TCR Repertoire in RdRp mRNA Vaccine Immunized Mice.

Effective T cell-targeting mRNA vaccines should raise sustained antigen-specific T cell responses. The persistence and functional phenotype of RdRp-specific CD8+ T cells in the peripheral blood of immunized C57BL/6 mice was tracked by flow cytometry. T cell responses elicited by a mRNA-iLNP vaccine encoding the SARS-CoV-2 Spike RBD were also monitored for comparison. The m1Ψ-modified, iLNP-encapsulated RBD mRNA construct tested in this experiment encodes amino acids 319 to 541 of the Wuhan strain SARS-CoV-2 Spike protein fused with the murine IgK signal sequence at the N terminus to improve protein secretion and the foldon trimerization domain of bacteriophage T4 at the C terminus to enhance antibody responses. This construct resembles the design of BNT162b1, which encodes the RBD of the SARS-CoV-2 Spike protein (SI Appendix, Fig. S2A) (29, 30). Spike RBD mRNA vaccine-encoded antigen expression was confirmed in transfected HEK293T cells (SI Appendix, Fig. S2B).

Mice were immunized with the RdRp mRNA vaccine, the Spike RBD mRNA vaccine, or control empty iLNP (iLNP) without RNA cargo on experiment days 0, 21, and 42 (Fig. 1*D*). Two cohorts of mice were immunized. For one cohort, lungs and spleen from mice were collected on day 9 following the second immunization to evaluate antigen-specific T cells. For the other cohort, the T cell response in peripheral blood of mice receiving three sequential immunizations was tracked longitudinally up to 91 d following the first immunization.

Total CD8+ T cell abundance in peripheral blood increased following RdRp or RBD mRNA vaccine immunization compared to mock immunization with empty iLNPs (SI Appendix, Fig. S2C). T cell expansion remained evident at 91 d following the first immunization (49 d after the third immunization). In contrast, immunization did not alter peripheral blood CD4+ T cell abundance (SI Appendix, Fig. S2C). To determine the specificity of T cells in immunized mice, PBMCs were restimulated ex vivo with RdRp or RBD peptide pools and evaluated by ICS of IFNγ and TNFα (Fig. 1*E*). Polyfunctional RdRp and RBD-specific CD8+ T cells were detected on day 7 following the second immunization. RdRp- and RBD-specific CD8+ T cell abundance in peripheral blood increased substantially following the third immunization (Fig. 1*E*). In this study, the RdRp vaccine induced a greater expansion of antigen-specific CD8+ T cells compared to the RBD vaccine. Despite some gradual contraction over time, a significant percentage of peripheral CD8+ T cells still exhibited reactivity to the RdRp or RBD peptide pools on day 91 following the first immunization (Fig. 1*E*). Considering that effector T cell populations contribute to the clearance of infected cells and the restriction of intrahost viral spread, the persistence of CD8+ T cells in immunized mice with an effector phenotype was defined. Most peripheral CD8+ T cells in immunized mice were defined as short-lived effector T cell (KLRG1+/CD127-) and effector (CD44+/CD62L-) phenotypes. A third immunization with either the RdRp or RBD vaccine further expanded the CD44+/CD62L- effector CD8+ T cell population (SI Appendix, Fig. S2D).

To determine whether the RdRp mRNA vaccine induces antigen-specific and effector phenotype T cells that circulate and traffic to nonlymphoid tissues, such as the lung, cells isolated from the lung and the spleen on day 9 following the second immunization were restimulated with the RdRp or RBD peptide pools and evaluated by IFNγ ELISpot (Fig. 1*F*). This result confirmed the presence of RdRp-specific T cells in these tissues. CD8+ T cells in the spleen exhibited an effector phenotype similar to the profile of peripheral blood CD8+ T cells (SI Appendix, Fig. S2E).

**Fig. 2.**
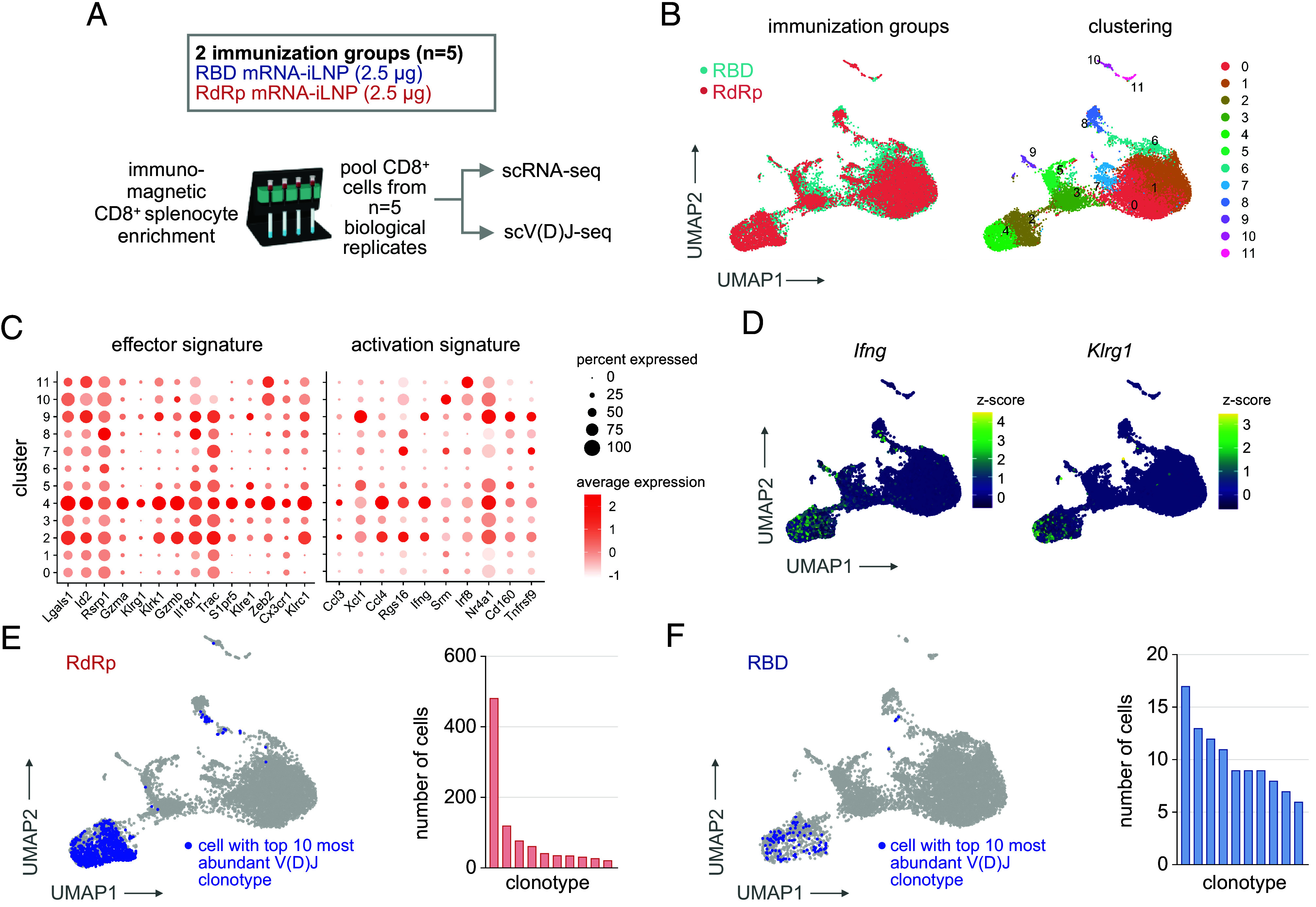
RdRp or RBD mRNA-iLNP immunization remodels the CD8+ T cell transcriptional landscape and TCR repertoire. (*A*) Single cell sequencing experiment to generate 5’GEX and V(D)J libraries of CD8+ splenocytes from mice immunized with two doses of RdRp or RBD mRNA-iLNP. Splenocytes from 5 animals/group on day 9 following boost immunization were pooled for enrichment and sequencing. (*B*) Uniform Manifold Approximation and Projection (UMAP) representation of CD8+ cell transcriptomes colored by experimental group or Louvain cluster assignment. (*C*) T cell effector or activation transcriptional signature enrichment across clusters. (*D*) Transcript level z-scores for representative T cell activation or effector genes. (*E*) UMAP representation and abundance of cells with the 10 most abundant TCR clonotypes in the RdRp mRNA-iLNP group. (*F*) UMAP representation and abundance of cells with the 10 most abundant TCR clonotypes in the RBD mRNA-iLNP group.

In a separate cohort of mice, single-cell sequencing was applied to define the transcriptional profile (5’GEX) and TCR repertoire of CD8+ splenocytes isolated from mice receiving primary and boost RdRp or RBD mRNA vaccine immunization (Fig. 2*A*) (31). CD8+ cells were clustered by single-cell gene expression profiles (Fig. *B*). Clustering identified specific populations of T cells exhibiting enrichment of T cell activation (*Ifng*) and T cell effector markers (*Klrg1*, *Gzma*, *Gzmb*) in both RBD and RdRp immunization groups (Fig. 2 *C* and *D*) (10). Analysis of V(D)J sequences indicated multiple high-frequency clonotypes (found in >5 cells) following RdRp or RBD vaccine immunization (Fig. 2 *E* and *F*). A greater number of CD8+ T cells in the RdRp group underwent clonal expansion compared to the RBD group. This is consistent with the fewer number of RBD-specific T cells compared to RdRp-specific T cells following 2 immunizations and the requirement for a third immunization for a substantial RBD-specific T cell expansion (Fig. 1*E*). Transcriptomics analysis highlighted that CD8+ T cells undergoing clonal expansion following immunization with either the RdRp or RBD vaccines were detected in clusters enriched for effector or activation transcriptional signatures (Clusters 2, 4 and Fig. 2 *C*, *E* and *F*) The single-cell transcriptional profile and TCR analysis indicated that both mRNA vaccines drive the clonal expansion of polyfunctional CD8+ T cells with an activated/effector transcriptional phenotype.

### T Cells Engineered to Express RdRp-Specific Murine TCRs Recognize a Conserved Epitope and Are Cytotoxic Against Cells Expressing RdRp.

**Fig. 3.**
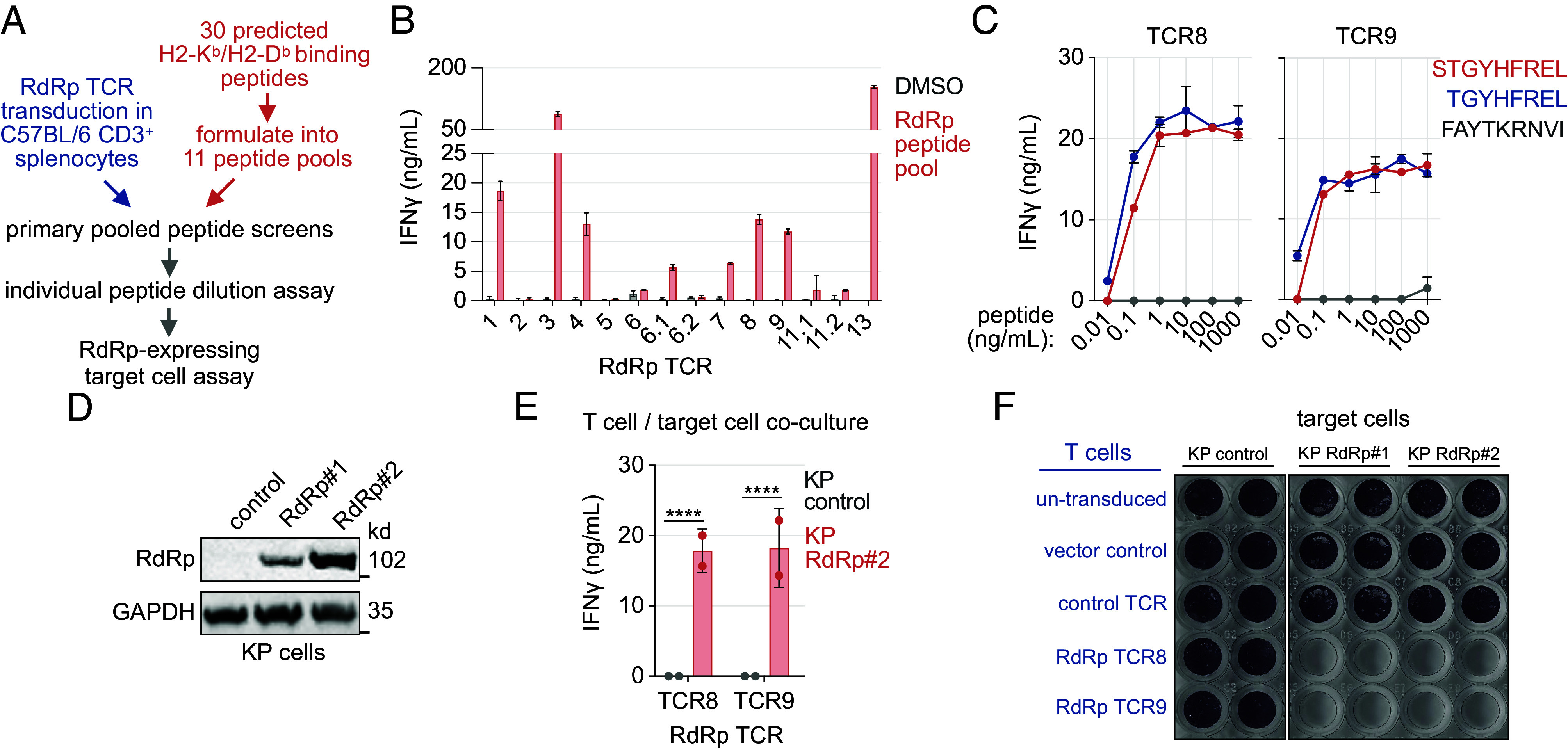
T cells expressing RdRp-specific TCRαβ recognize a conserved epitope and kill RdRp-expressing cells. (*A*) Strategy for the deconvolution of peptide antigens recognized by T cells undergoing clonal expansion in RdRp mRNA-iLNP immunized mice. (*B*) IFNy ELISA analysis of TCRαβ-transduced CD3+ splenocytes restimulated with a RdRp peptide pool (mean ± SD; n = 2 biological replicates). (*C*) IFNγ ELISA analysis of TCRαβ-transduced CD3+ splenocytes restimulated with a dose range of RdRp peptides (mean ± SD; n = 2). (*D*) Immunoblot analysis of murine KP4662 (KP) cells engineered to stably express trRdRp. (*E*) IFNγ ELISA analysis of RdRp TCRαβ-transduced CD3+ splenocytes co-cultured with RdRp-expressing KP cells at a 8:1 effector:target (E:T) ratio for 48 h (mean ± SEM; n = 2; unpaired *t* test). (*F*) Crystal violet cell survival analysis of adherent RdRp-expressing KP cells co-cultured with TCRαβ-transduced or control T cells at an 8:1 effector:target ratio for 72 h (n = 2). **** *P* < 0.0001.

The presence of high-frequency clonotypes following RdRp mRNA vaccine immunization, such as the most abundant clonotype with >400 cells, could indicate that the vaccine encodes immunodominant epitope(s). The immunodominance of RdRp epitopes has been demonstrated in human CD8+ T cells targeting RdRp (11). To define potential immunodominant epitopes encoded by the RdRp vaccine, the epitope specificity of high-frequency TCRs detected in immunized mice was evaluated (Fig. 2*E*). 14 TCR alpha and beta chains corresponding to the most abundant clonotypes detected by single-cell sequencing were expressed in C57BL/6 CD3+ splenocytes by retroviral transduction (Fig. 3*A*). CD3+ cells expressing 10 of the 14 cloned TCRs were reactive to stimulation with the RdRp peptide pool, as indicated by IFNγ production (Fig. 3*B*). The TCRs were screened against a custom library of 30 unique 8-11mer RdRp peptides encoded by the mRNA construct. These peptides were chosen for evaluation based on binding affinity prediction for mouse H2-K^b^ and H2-D^b^ MHC I molecules by NetMHCpan4.1 (32). For epitope deconvolution, cloned TCRs were screened for reactivity against 11 subpools, each containing 5 to 6 individual RdRp peptides (SI Appendix, Fig. S3A). Each candidate peptide was included in 2 unique subpools. TCR-engineered CD3+ cells were stimulated with peptide subpools, and epitope specificity was indicated by IFNγ production measured by ELISA (SI Appendix, Fig. S3B). All tested TCRs that were reactive to the 15mer RdRp sliding window peptide pool were also reactive to peptide subpools containing 8mer and 9mer (S)TGYHFREL peptides that are predicted to bind H2-K^b^ or H2-D^b^, respectively. The (S)TGYHFREL peptides at a 1 ng/mL concentration potently stimulated IFNγ production by TCR8 and TCR9-expressing CD3+ cells (Fig. 3*C*). No further significant increase in IFNγ production was observed when higher peptide concentrations were tested. Another RdRp peptide predicted to bind H2-D^b^, FAYTKRNVI, elicited a weak IFNγ response at a much higher concentration of 1 µg/mL (Fig. 3*C*). Our analysis revealed that TCR clonotypes expanded by the RdRp mRNA vaccine in C57BL/6 mice recognize a single RdRp epitope shared by SARS-CoV-2 variants. The results from the RdRp-specific TCR characterization are summarized in SI Appendix, Fig. S3C.

Clearance of infected cells to reduce viral replication contributes to the protection afforded by CD8^+^ T cells. Therefore, the cytotoxicity of T cells expressing RdRp-specific TCRs against target cells expressing RdRp was evaluated. CD3+ cells expressing RdRp-specific TCRs were cocultured with 2 clones of a murine cell line (KP) engineered to express the truncated form of RdRp encoded by the vaccine (Fig. 3*D*). KP cells were stimulated with murine type I IFN (IFNβ) for 72 h before coculture to elevate cell surface MHC I levels (SI Appendix, Fig. S3D). IFNγ production by RdRp TCR8- and TCR9-expressing T cells was observed following culture with target cells expressing RdRp but not control cells (Fig. 3*E*). The recognition of RdRp-expressing target cells by T cells expressing TCR8 and TCR9 indicated the presentation of (S)TGYHFREL by MHC class I on target cells and recognition by RdRp-specific TCRs. The survival of adherent KP cells following coculture was evaluated to assess the cytotoxicity of RdRp-specific TCR-engineered T cells. The two target cell clones expressing RdRp, but not control cells, were eradicated by T cells expressing RdRp-specific TCR8 and TCR9 after 72 h of coculture (Fig. 3*F*). CD3+ splenocytes that were mock-transduced or transduced with an irrelevant pmel-1 TCR that targets the murine melanoma antigen gp100 did not exhibit cytotoxic activity against any tested target cells (33). These data indicate that i) murine T cells engineered to express RdRp-specific TCRs recognizing and kill RdRp-expressing cells, and ii) that the immunodominant RdRp epitope (S)TGYHFREL can be processed and presented by MHC I in murine cells. RdRp- and Spike RBD-encoding mRNA vaccines elicit CD8+ T cell responses specific to conserved immunodominant epitopes in C57BL/6 mice. Having defined CD8+ T cell epitopes, and as CD4+ T cell help is necessary for optimal CD8+ T cell responses, we performed in silico analysis to confirm the presence of CD4+ T cell epitopes encoded by the RdRp mRNA vaccine (SI Appendix, Fig. S3E).

To determine the extent to which CD8+ T cells elicited by RdRp mRNA vaccine immunization in mice recognize the STGYHFREL epitope, reactivity to this peptide in immunized mice was evaluated by IFNγ ELISpot analysis of cells isolated from spleen and lung (SI Appendix, Fig. S4A). Previously, Spike-specific CD8+ T cell epitopes were identified in infected C57BL/6 mice (34). Among those encoded by the RBD mRNA vaccine, responses to the VVVLSFEL (S510 to 517) epitope are the most dominant. The Spike RBD VVVLSFEL epitope is conserved across all described SARS-CoV-2 variants and is H2-K^b^ restricted. T cell responses to this epitope were analyzed in mice receiving the RBD mRNA vaccine. Splenocytes and lung cells were isolated from mice immunized with the RdRp or RBD vaccine on day 9 following the second immunization (as in Fig. 1*D*) and restimulated with either the RdRp STGYHFREL peptide or the RBD VVVLSFEL peptide. Strong epitope-specific responses were observed (SI Appendix, Fig. S4A). IFNγ and TNFα ICS analysis of PBMC restimulated with the RBD peptide or RdRp peptide was also performed (SI Appendix, Fig. S4B). A substantial fraction of CD8+ T cells from each group of immunized mice at day 49 and 91 after the first immunization (corresponding to day 7 and 49 after the third immunization) are specific for respective peptides (SI Appendix, Fig. S4B). These data indicate that RdRp and RBD mRNA vaccines elicit strong CD8+ T cell responses specific to highly conserved SARS-CoV-2 epitopes.

### Simultaneous RBD and RdRp Vaccine Administration Suppresses RBD-Specific T Cell and Antibody Responses.

**Fig. 4.**
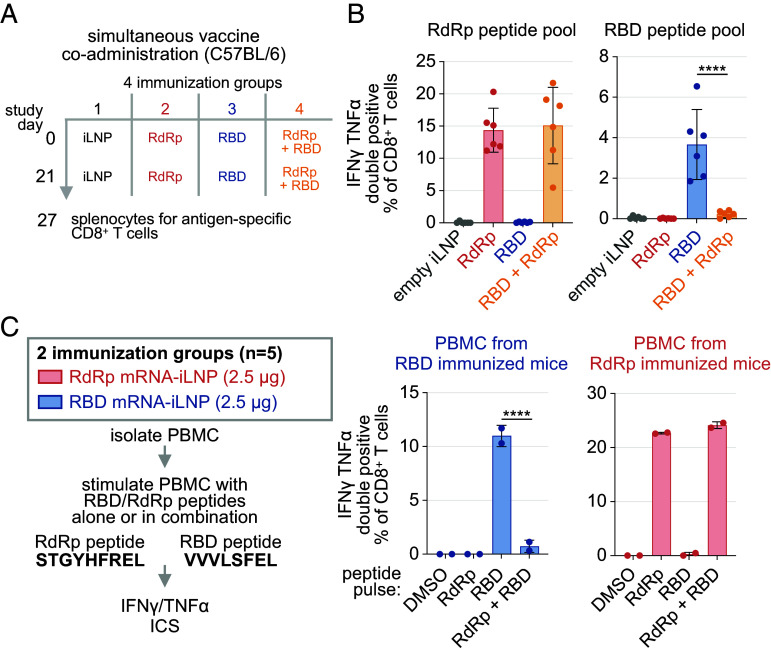
Simultaneous RdRp and RBD mRNA vaccine immunization diminishes spike RBD-specific CD8+ T cell responses. (*A*) Experimental design to monitor immune response in C57BL/6 mice immunized with RBD and RdRp mRNA-iLNP individually or simultaneously (2.5 μg; i.m.). (*B*) ICS analysis of day 27 splenocytes from experiment in Fig. 4*A* pulsed with RdRp or RBD peptide pools (mean ± SD; n = 5; one way ANOVA). (*C*) ICS analysis of PBMC from mice immunized with RdRp or RBD mRNA-iLNP as in Fig. 1*D* restimulated with RdRp (STGYHFREL) or RBD (VVVLSFEL) peptides alone or together (0.25 μg/mL). PBMC were obtained from mice immunized with three doses of 2.5 μg RdRp or RBD mRNA-iLNP. PBMC from 5 immunized mice from each group were pooled before ex vivo peptide restimulation, ICS analysis, and flow cytometry. Replicates represent individual stimulations (wells) of pooled PBMC (mean ± SD; n = 2; one way ANOVA). **** *P* < 0.0001.

Multiple antigens can be combined in one immunization to increase the breadth of T cell and antibody responses (10). Coadministration with mRNA vaccines encoding distinct epitopes introduces the challenge of preserving the immunogenicity of each vaccine. The impact of coadministration on the immunogenicity of RdRp and RBD vaccines was defined. The two mRNA-iLNP were combined 1:1 and coadministered to WT C57BL/6 mice via a single intramuscular injection (Fig. 4*A*). IFNγ ELISpot and flow cytometry ICS analysis of splenocytes following peptide pool restimulation indicated that RdRp-specific T cell responses at day 6 after the second immunization were comparable between mice receiving the RdRp vaccine alone and in combination with the RBD vaccine (Fig. 4*B* and SI Appendix, Fig. S4C). In contrast, the generation of RBD-specific CD8+ T cell responses was suppressed in cohorts receiving the vaccine combination compared to the cohort receiving the RBD vaccine alone (Fig. 4*B* and SI Appendix, Fig. S4C). This experiment unexpectedly revealed that coadministration of the RdRp vaccine with the RBD vaccine suppresses RBD-specific CD8+ T cell responses.

The dampening of RBD-specific T cell responses in mice receiving a mixture of the two vaccines could result from antigen-specific competition between mRNA-encoded epitopes for presentation by APCs, or epitope-independent vaccine interference. Antigenic competition impacts immunodominance and the generation of antigen-specific T cell responses to infection (35). To test the hypothesis of antigenic competition, we examined whether the vaccine-encoded RdRp immunodominant epitope affects the ability of the RBD epitope to activate T cells during ex vivo restimulation. Isolated PBMC from mice immunized individually with RBD or RdRp mRNA vaccines were restimulated ex vivo with the immunodominant RdRp or RBD peptide alone or together (Fig. 4*C* and SI Appendix, Fig. S4D). T cell activation was monitored by ICS analysis of IFNγ and TNFα. The addition of the RBD VVVLSFEL peptide had no impact on the ability of the RdRp STGYHFREL peptide to stimulate cytokine responses in RdRp-specific T cells. On the other hand, the addition of the RdRp peptide potently suppressed RBD peptide-mediated stimulation of RBD-specific T cells (Fig. 4*C* and SI Appendix, Fig. S4D). These results indicate that peptide epitopes encoded by different mRNA vaccines, such as RBD and RdRp, may compete for presentation when delivered as a mixture, diminishing the resulting T cell responses compared to individual administration. Antigenic competition for the stimulation of T cell responses may be MHC haplotype-specific and linked to specific vaccine-encoded immunodominant epitopes.

The presentation likelihood of the immunodominant epitopes encoded by each vaccine was defined in silico using the NetMHCpan4.1-BA and NetMHCpan4.1-EL algorithms within the Next-Generation Immune Epitope Database and Tools class I T cell prediction analysis resource (36). The -BA algorithm predicts the strength of the peptide:MHC interaction, while the -EL algorithm considers natural antigen processing pathways in addition to the strength of this interaction. We compared the percentile ranks for each peptide presented by either H2-K^b^ or H2-D^b^. The -EL percentile rank for each peptide:MHC pair was generated by comparing their predicted elution score against a set of random peptides. The -BA percentile ranks were calculated similarly, utilizing their predicted IC50 binding value instead. The RdRp H2-K^b^ restricted peptide identified by our studies, TGYHFREL, ranked among the top 0.01% of all peptides whereas the RBD peptide, VVVLSFEL, ranked among the top 0.2% of peptides using the -EL algorithm. In addition to the strong presentation probability on H2-K^b^, the 9-mer RdRp peptide, STGYHFREL, falls within the top 2% of presented peptides on H2-D^b^, unlike the RBD peptide VVVLSFEL. This result indicated that the 8mer RdRp epitope TGYHFREL and the 8mer RBD VVVLSFEL epitopes are H2-K^b^-restricted while the 9mer RdRp STGYHFREL epitope is predicted to bind H2-D^b^. Although the STGYHFREL peptide was used in Fig. 4*C*, N-terminal trimming may generate the TGYHFREL epitope. This analysis, and the observed suppression of RBD-specific T cell responses to the VVVLSFEL peptide when the STGYHFREL peptide is codelivered, provide evidence for antigenic competition (SI Appendix, Fig. S4E).

**Fig. 5.**
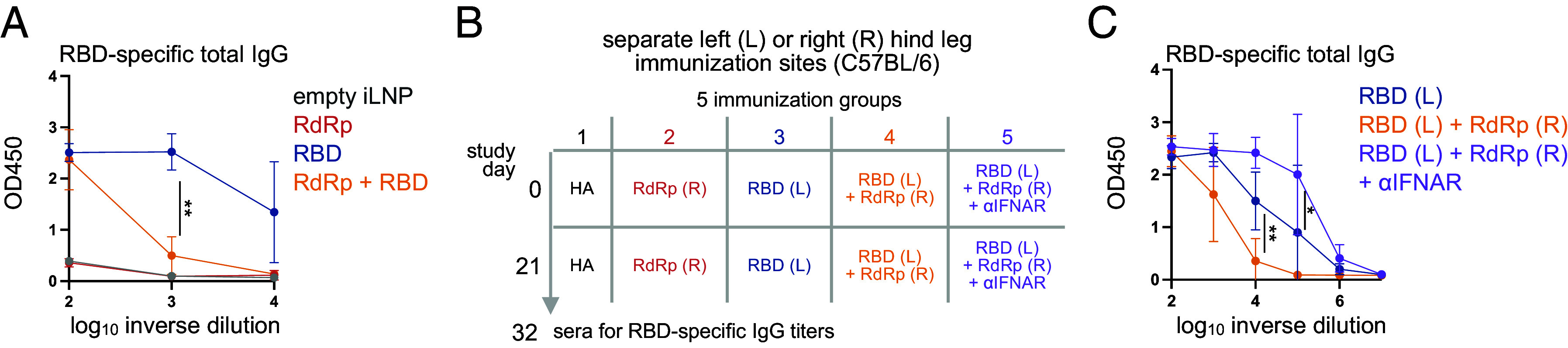
Type I IFN signaling restrains spike RBD-specific antibody responses elicited by immunization. (*A*) ELISA analysis of sera Spike S1-specific IgG titers at the endpoint from experiment in Fig. 4*A* (mean ± SD; n = 5; one way ANOVA). (*B*) Experimental design to monitor immune response in C57BL/6 mice immunized with RBD or RdRp mRNA-iLNP individually at separate *Left* (L) or *Right* (R) gastrocnemius muscle injection sites (2.5 μg; i.m.). A non-SARS-CoV-2-targeting mRNA-iLNP encoding hemagglutinin of influenza A virus (HA) was administered as a control. IFNAR-blocking or isotype control antibodies were administered by i.p. injection 1 d before mRNA vaccine immunization. (*C)* ELISA analysis of serum Spike S1-specific IgG titers at the experimental endpoint (mean±SD; n = 3 to 4; one way ANOVA).* *P* <0.05; ** *P* <0.01.

To determine whether vaccine coadministration with RdRp and RBD vaccines impacts the generation of Spike RBD-specific IgG responses, the sera of mice receiving two doses of each vaccine or the combination from the experiment in Fig. 4*A* was evaluated using a Spike S1-specific ELISA. While the RBD vaccine elicited a potent RBD-specific IgG response in sera at day 6 following boost immunization, serum RBD-specific IgG titers were potently suppressed in the coadministration group (Fig. 5*A*). No RdRp-specific antibodies were detected in an RdRp-specific ELISA analysis of the same sera samples.

To mitigate the potential competition for presentation between epitopes encoded by RdRp and RBD mRNA during coadministration, the simultaneous contralateral administration of the vaccines in either the left or right gastrocnemius muscle was tested (Fig. 5*B* and SI Appendix, Fig. S4F). In our separate study on the RBD vaccine in C57BL/6 mice, blocking IFN responses by type I IFN receptor (IFNAR) neutralizing antibodies significantly increase the RBD-specific antibody response. Therefore, the role of type I IFN in antibody suppression during coimmunization was also tested in this contralateral administration by administering an IFNAR-blocking antibody 1 d prior to immunization (Fig. 5*B*). Contralateral administration largely restored RBD-specific T-cell responses as measured by ICS and ELISpot analysis of splenocytes (SI Appendix, Fig. S4F). In contrast, serum Spike-binding antibodies 11 d after the second immunization remained suppressed following contralateral immunization (Fig. 5*C*). Notably, IFNAR blockade alleviated the suppression of the Spike-binding IgG response and increased sera Spike-specific IgG to a level higher than observed with RBD vaccine immunization alone (Fig. 5*C*). The data suggest that multiple mechanisms of vaccine interference following coadministration —antigen-specific epitope competition and type I IFN signaling—contribute to the negative effects on RBD-specific T cell and antibody responses.

### Staggered RdRp mRNA Vaccine Administration Maintains Spike RBD Vaccine-Driven Immunogenicity and SARS-CoV-2 Protection.

**Fig. 6.**
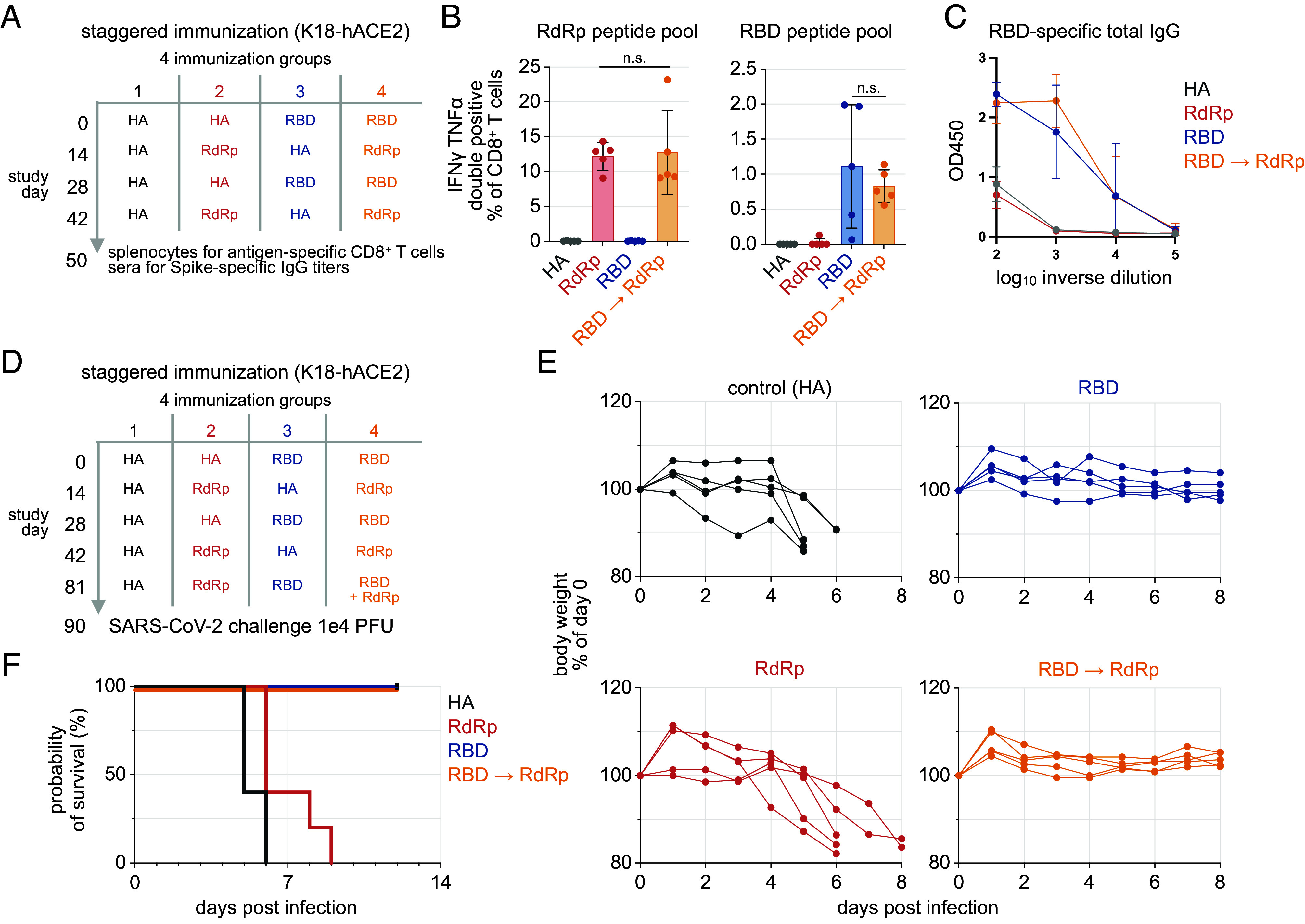
Staggered RdRp vaccine immunization preserves the immunogenicity and protection from SARS-CoV-2 challenge conferred by the RBD vaccine. (*A*) Experimental design to test the immunogenicity of RdRp and RBD mRNA-iLNP following staggered immunization in K18-hACE2 C57BL/6 mice (2.5 μg; i.m.). A non-SARS-CoV-2-targeting mRNA-iLNP encoding HA was administered as a control. (*B*) ICS and ELISpot analysis of day 50 splenocytes pulsed with RdRp or RBD peptide pools (mean ± SD; n = 5; one way ANOVA). (*C*) ELISA analysis of serum Spike S1-specific IgG titers at the experimental endpoint (mean ± SD; n = 5). (*D*) SARS-CoV-2 lethal challenge in RdRp, RBD or HA mRNA-iLNP immunized K18-hACE2 C57BL/6 mice (1e4 PFU SARS-CoV-2; 2.5 μg mRNA vaccine; i.m.; n = 5). (*E*) Body weight change following challenge. (*F*) Survival of mice following 1e4 PFU SARS-CoV-2 challenge. n.s.: not significant.

The observed competition between the RBD and RdRp epitopes encoded by the vaccines highlights a limitation of simultaneous coadministration for expanding the breadth of CD8+ T cell responses. As contralateral administration did not fully restore the Spike-specific T cell or IgG responses, a temporally staggered immunization schedule where mice received RdRp and RBD mRNA vaccines with a 2-wk space between each immunization was evaluated (Fig. 6*A*). This timepoint was chosen to mitigate vaccine interference due to type I IFN signaling and epitope competition as the expression of proteins encoded by LNP-encapsulated, nucleoside-modified mRNA following intramuscular injection can be sustained for 10 d following immunization (37). Mice expressing the human angiotensin-converting enzyme 2 (hACE2) driven by the keratin 18 promoter (K18-hACE2) were utilized for this experiment (38). The K18-hACE2 model allows for SARS-CoV-2 infection in mice that are otherwise not susceptible (38). ICS and ELISpot analysis of splenocytes indicated no difference in the magnitude of RBD- or RdRp-specific CD8+ T cells across groups of mice receiving each vaccine alone or in combination (Fig. 6*B*). However, the levels of RBD-specific CD8+ T cells following immunization were lower in K18-hACE2 mice compared to wild-type C57BL/6 mice. The staggered immunization schedule also preserved Spike S1-specific IgG responses as measured by ELISA (Fig. 6*C*). The staggered coadministration enables the generation of strong T cell responses against both Spike and RdRp epitopes.

A third mRNA vaccine dose increases the abundance of antigen-specific CD8+ T cells and a third immunization with a single immunodominant Spike T cell epitope (S539 to 546) has been shown to be required to prevent the lethality SARS-CoV-2 infection in K18-hACE2 mice (39).

Three RBD or RdRp vaccine doses alone or in combination were administered to K18-hACE2 mice using the staggered immunization schedule (Fig. 6*D*). Mice were challenged 9 d after the final immunization with 1 × 10^4^ PFU of WA1 strain SARS-CoV-2. Compared to the control group, which demonstrated a median survival of 5 d, the RdRp vaccine provided moderate survival benefits with a median survival of 6 d (Fig. 6*E*). Notably, the cohorts of mice receiving the RBD vaccine alone or a combination of RBD and RdRp vaccines were protected with no measurable weight loss (Fig. 6*F*).

K18-hACE2 mice receiving two or three RBD vaccine doses developed Spike-binding IgG antibodies detectable in sera (SI Appendix, Fig. S5A). There was no difference in Spike-specific IgG antibodies between mice immunized with the RBD vaccine and those coimmunized with RBD and RdRp vaccines. The levels of Spike-specific IgG in sera were not significantly different in mice receiving two or three immunizations with the RBD vaccine (SI Appendix, Fig. S5A). Spike-specific total IgG levels were lower in K18-hACE2 mice receiving either two or three RBD vaccine doses compared to Balb/c strain mice receiving two doses (SI Appendix, Fig. S5A). This difference may be due to the C57BL/6 and BALB/c strains modeling Th1- and Th2-type responses (40). While immunized mice possessed Spike-specific IgG, SARS-CoV-2 neutralizing antibodies in sera from mice receiving three vaccine doses were below the detection limit (SI Appendix, Fig. S5B).

The results indicate that Spike RBD vaccine immunization in C57BL/6 mice provides protection against lethal SARS-CoV-2 challenge in the absence of neutralizing antibodies. They also indicate that protection can be maintained with RdRp mRNA vaccine coadministration using a strategic staggered immunization approach that preserves the T cell and antibody responses elected by each vaccine component.

## Discussion

Our studies indicate that a nucleoside-modified mRNA vaccine encoding the SARS-CoV-2 RdRp protein raises antigen-specific CD8+ T cell responses in wild-type and transgenic mice expressing a common human MHC I allele, HLA-A2.1 (38). T cells expressing TCRs identified in cells clonally expanded by the RdRp vaccine recognize and kill RdRp-expressing target cells. Both RBD and RdRp mRNA vaccines are highly immunogenic, and ~20% of peripheral blood of CD8+ T cells are antigen-specific at day 49 after the third immunization. The most unexpected outcome of the experiments was the finding that the coadministration of two mRNA vaccines encoding RdRp and Spike RBD antigens dampened RBD-specific T cell and antibody responses. We provide evidence that suppression can be attributed to antigenic competition for T cell responses and type I IFN signaling for antibody responses. A staggered immunization strategy avoids suppression to enhance the breadth of mRNA vaccine-driven immune responses.

RdRp is one of the six main viral antigen targets of T cell responses in infected individuals. RdRp-specific T cells are considered moderate in abundance compared to Spike and Nucleocapsid-targeting responses (12, 13). Our results indicate that RdRp specific T cells may be expanded through immunization with an mRNA-iLNP vaccine encoding the N terminus of RdRp optimized for T cell responses (23, 41). Protection was assessed in the K18-hACE2 transgenic mouse model, where SARS-CoV-2 infection leads to lethality and severe disease that includes neuroinvasion (42–44). Immunization with the RdRp mRNA vaccine provided a modest survival benefit but did not prevent death (Fig. 6*F*). We put forth two possible explanations for the failure of RdRp-specific T cells alone to protect transgenic K18-hACE2 mice against SARS-CoV-2. The first relates to the nonphysiological overexpression and ectopic expression of the viral entry receptor due to the cytokeratin K18 promoter used in this model. In an alternative transgenic mouse model where hACE2 is inserted immediately downstream of the translational initiation codon of endogenous ACE2, SARS-CoV-2 replicates less and generates more than 1,000-fold fewer progeny virions compared to K18-hACE2 mice without causing death (45). In K18-hACE2 mice, RdRp-specific T cells may not act rapidly enough to halt the production of virions by infected cells. The second is insufficient levels of RdRp peptide–MHC I complexes on the surface of infected cells, which limits the protective efficacy of T cell-mediated killing. Mass spectrometry proteomics indicated that RdRp protein levels are much lower than structural proteins such as Spike and Nucleocapsid during viral replication (46). While the RdRp mRNA vaccine does elicit robust RdRp-specific responses, low RdRp expression in infected cells may hinder efficient recognition and killing by RdRp-specific T cells in the K18-hACE2 mouse model.

In contrast, complete protection against lethal infection was observed in cohorts of mice receiving three doses of the RBD vaccine alone or in combination with the RdRp vaccine. Although we did not detect neutralizing antibodies in these protected mice prior to challenge, as the RBD mRNA vaccine elicits a robust CD8+ T cell response, it may protect through T cells. Moreover, we demonstrated that a third immunization significantly increased the abundance of RBD-specific CD8+ T cells (Fig. 1*E*), which may explain the necessity of a third immunization for protection. Our data are similar to a published study showing that a third immunization with a single immunodominant Spike T cell epitope (S539 to 546) is required to prevent the lethality SARS-CoV-2 infection in K18-hACE2 mice (39). However, our RBD vaccine does not contain the S539 to 546 epitope and instead elicits CD8+ T cells primarily targeting the S510 to 517 epitope, which is also one of the more dominant epitopes identified in SARS-CoV-2-infected C57BL/6 mice (34). Nonetheless, we cannot exclude the possibility of protection through antibody-dependent cellular cytotoxicity or other Fc-related mechanisms. Future studies are warranted to elucidate the contribution of these immune mechanisms to the protection observed with our RBD vaccine.

Coadministration of multiple vaccines can increase the efficacy of protection but introduces the challenge of preserving the immunogenicity of each vaccine. In humans, a diminished response to a vaccine antigen can sometimes be observed when administered as part of a multivalent vaccine compared to when given alone (47). Understanding the mechanisms underlying diminished responses can guide the development of future multivalent vaccines to expand the repertoire of vaccine-driven T cell and antibody responses. Our studies provide an example of negative effects resulting from the administration of two iLNP-encapsulated mRNA vaccines together in mice, as well as a mitigating strategy. When RBD and RdRp mRNA-iLNP were mixed and administered as a single injection, RBD-specific antibody and T cell responses were significantly reduced. In contrast, RdRp-specific T cell responses were unaffected compared to the administration of the individual vaccine. However, a temporally staggered immunization regime preserves the immunogenicity of both vaccines.

The vaccine interference we observed may be driven by systemic or local cytokine responses to vaccine components, such as iLNP and RNA cargo (47). In humans and mice, commercial SARS-CoV-2 encoding mRNA vaccines trigger potent inflammatory responses and drive a transient elevation in the systemic levels of the cytokine type I IFN (48). In a separate study, we showed that RBD and RdRp mRNA vaccines induce greater type I IFN responses compared to the administration of empty iLNP. The in vitro transcribed mRNA used to formulate the mRNA-iLNP tested in this study was not subjected to HPLC- or cellulose-based purification to remove dsRNA by-products that could trigger type I IFN responses (49). Type I IFN signaling has been shown to dampen the translation of vaccine-encoded mRNA to limit antigen expression (48). Previous studies have established that ablating type I IFN signaling improves antibody responses in settings of viral infection and viral vaccines (50–53). A similar improvement of IFN signaling blockade on RBD-specific antibodies was observed during contralateral administration of RBD and RdRp mRNA-iLNP (Fig. 5*C*). Thus, reduced RBD-specific antibodies observed during coadministration with RdRp mRNA-iLNP are likely due to higher IFN levels driven by the double amount of mRNA-LNP compared to individual immunizations. We cannot determine whether such vaccine interferences affect both antigens in coadministration since RdRp mRNA-iLNP did not induce antibody responses.

Our studies provide evidence that antigenic competition contributes to the suppression of RBD-specific T cell responses following coadministration. In in vitro experiments testing PBMC obtained from mice immunized with the RBD vaccine alone, we demonstrated that the addition of the immunodominant CD8+ T cell RdRp epitope peptide potently suppresses the activation of CD8+ T cells by the immunodominant RBD epitope peptide (Fig. 4*C*). We postulate this result as indicating that the RdRp epitope peptide exhibits a higher affinity for the C57BL/6 MHC I molecules compared to the RBD epitope. This is supported by an in silico analysis that predicted a strong RdRp peptide:MHC interaction (SI Appendix, Fig. S4E).

The interference of the RdRp peptide in the ex vivo restimulation experiment implies that during priming, the RdRp epitope may interfere with the RBD epitope for binding to MHC class I molecules of APC, thereby reducing the activation of RBD-specific T cells during coadministration. Consistent with this notion, when the two vaccines were administered via separate contralateral injection sites, RBD-specific T cell responses were no longer significantly impacted (SI Appendix, Fig. S4F). In addition, separating the administration of the two vaccines by 2 wk was sufficient to overcome the negative effects on T cell responses (Fig. 6 *A* and *B*). Such vaccine interferences caused by epitope competition are likely antigen-specific and depend on the binding affinity of each epitope to MHC class I molecules, the stability of each peptide–MHC complex, and the interactions between the peptide–MHC complexes and respective TCR molecules. In conclusion, mitigating epitope competition and IFN-mediated vaccine interference, as described here, should be considered in the development of multivalent mRNA vaccines against future SARS-CoV-2 variants, other infectious agents, or cancer.

### Limitations of the Study.

Protection against the WA1 SARS-CoV-2 strain was not provided by the RdRp vaccine alone in the murine K18-hACE2 model. Whether RdRp-encoding vaccines confer protection, alone or in combination with Spike-encoding vaccines in other more relevant preclinical challenge models such as antibody-resistant SARS-CoV-2 variants or the Syrian Hamster model, which relatively recapitulates the immunopathology of SARS-CoV-2 infection in humans, has not yet been determined (54). As protection against WA1 SARS-CoV-2 was observed in cohorts receiving the RBD vaccine alone but not in cohorts receiving the RdRp vaccine alone, we infer that RBD-specific responses primarily mediate the protection observed when both vaccines were administered. This conclusion could be further strengthened by directly comparing the protection between staggered immunization, which preserves both RBD-specific antibody and T cell responses, and simultaneous immunization, where RBD-specific T cell and antibody responses are suppressed.

## Methods

### Cell Culture.

HEK293T, B16F10, and KP4662 cells were cultured in Dulbecco’s Modified Eagle’s Medium (DMEM; Corning #10-017-CV) supplemented with 10% fetal bovine serum (FBS; Omega Scientific) and 1X Penicillin/Streptomycin (Thermo Fisher Scientific #15140122; complete DMEM). Cells were maintained between passages 3 and 20 at 37 °C and 5% CO_2_. HEK293T cells were purchased from ATCC (#CRL-3216). VERO C1008 (VeroE6) cells were purchased from ATCC (#CRL-1586TM). KP4662 (Kras^G12D/+^; Tp53^R172H/+^) murine pancreatic cancer cells derived from tumors formed in transgenic mice were a gift from Robert Vonderheide at the University of Pennsylvania (Philadelphia, PA). Cell cultures were routinely monitored for *mycoplasma* contamination by luminescence-based detection (Lonza #LT07-118).

### Mouse Models and Treatment.

All mouse experiments were conducted with the approval of the UCLA Institutional Animal Care and Use Committee, known as the Chancellor’s Animal Research Committee (Protocols: ARC-2010-140 and ARC-2007-149); and the USC institutional Animal Care and Use Committee. Female 8 to 10 wk old C57BL/6 J, HLA-A2.1, K18-hACE2, and Balb/c mice were purchased from the Jackson Laboratory (#000664, #004191, #034860, and #000651 respectively). For immunizations, mice were cleaned with isopropanol wipes, and administered 50 µL of mRNA-iLNP diluted in sterile PBS via intramuscular (i.m.) injection in the hindleg (gastrocnemius muscle) with an insulin syringe (BD #329461). IFNAR neutralizing antibody (BioXCell #BE0241) and IgG1 isotype control antibody (BioXCell #BE0083) were administered by intraperitoneal (i.p.) injection. All experiments involving mice were performed according to the NIH guidelines for the care and use of laboratory animals.

### mRNA Vaccine Design, Synthesis, and Lipid Nanoparticle Encapsulation.

mRNA sequences described in the text were codon optimized to minimize uridine content with Geneious software (Biomatters Inc.) and synthesized using N1-Methylpseudouridine-5’-Triphosphate (m1Ψ-UTP) and 5’ CleanCap with silica-membrane purification by TriLink Biotechnologies. Lipid nanoparticles were prepared by Acuitas Therapeutics using a self-assembly process (25). The ionizable cationic lipid-containing nanoparticle formulation used in this study is proprietary to Acuitas Therapeutics; the proprietary lipid and the lipid nanoparticle composition is described in U.S. patent no. US10221127. The mRNA-containing lipid nanoparticles were characterized and subsequently stored at −80 °C at an RNA concentration of 1 mg/mL.

### SARS-CoV-2 Culture for Challenge Studies.

Vero E6 cells overexpressing ACE2 (VeroE6-hACE2) were obtained from Dr. Jae Jung (USC) and maintained in DMEM high glucose, supplemented with 10% FBS and 2.5 μg/mL puromycin at 37 °C in a humidified atmosphere at 5% CO_2_. SARS-CoV-2 virus (BEI #NR-52281) was cultured and VeroE6-hACE2 infected. A singular plaque isolate was collected and passaged 4 times in VeroE6-hACE2 cells and harvested 48 h postinoculation each time. Plaque forming units (PFU) were determined by plaque assay. A monolayer of VeroE6-hACE2 cells was infected with a serial dilution of virus stocks and overlay of semisolid agar. At day 3 postinfection, plaques were fixed with 4% paraformaldehyde. Plaque visualization was performed by removing the agarose layer and staining with 0.2% (w/v) crystal violet solution. Plaques were counted to determine PFU. Virus stocks were stored at −80 °C.

### SARS-CoV-2 Challenge.

All challenge studies were performed entirely by the University of Southern California COVID Core team at the Hastings Foundation and Wright Foundation BSL3 facility. During the immunization period prior to challenge, K18-hACE2 mice were housed in a BSL2 facility. Prior to SARS-CoV-2 challenge, mice were transferred to ABSL3 containment facility. Mice were administered 1 × 10^4^ PFU of WA1 strain SARS-CoV-2 cultured as described above in 30 µL of PBS, by intranasal inoculation. Mice were monitored, weighed daily and sacrificed following approved humane end points or set end point at day 5 post infection. Humane end points included, but were not limited to, failure of reflex tests, or greater than 20% prechallenge body weight loss.

### In Vitro Viral Inhibition Assay.

SARS-CoV-2 viral inhibition assays to assess serum Spike S1 neutralizing antibody levels were performed at the UCLA BSL3 high containment facility. For sera isolation, blood from immunized mice was collected in 1.5 mL Eppendorf tubes by the retro-orbital technique using heparin-coated capillary tubes, incubated at 56 °C for 30 min to inactivate the complement and centrifuged at 2,000×*g* for 15 min at 4 °C. Serum supernatants were transferred to a new tube and stored at −80 °C before analysis. VeroE6 (VERO C1008) cells were cultured at 37 °C with 5% CO_2_ in EMEM growth media with 10% FBS and 100 units/mL penicillin. SARS-CoV-2 Isolate USA-WA1/2020 was obtained from BEI Resources of National Institute of Allergy and Infectious Diseases. VeroE6 cells were plated in 96-well plates (5 × 10^3^ cells/well). Serially diluted (twofold) mouse sera was incubated with virus (100 PFU/well) for 1 h at room temperature prior to addition to cells. At 48 h postinfection, the cells were scored for presence or absence of SARS-CoV-2 mediated cytopathic effect. The wells showing viral inhibition at the highest dilution of each sera sample is considered for calculating titer.

### Immunoblot Analysis.

1.5 × 10^5^ 293 T cells were seeded overnight in 0.5 mL culture medium in a 24-well plate (VWR #10062-896). The next day, culture medium was changed to media containing 0.5 µg mRNA-iLNP. 24 h post transfection, cells were lysed in RIPA buffer containing 1 mM phenylmethylsulfonyl fluoride for 30 min at 4 °C. 4× Laemmli buffer (Bio-Rad #1610747) with 10% beta-mercaptoethanol was added to lysates and samples were boiled at 95 °C for 15 min. Lysates were run on a homemade 10% SDS-PAGE gel or a precast NuPage 4 to 12% gel (Thermo #NP0336) alongside a 10 to 250 kDa molecular weight ladder (Thermo Fisher Scientific #26619). After proteins were transferred to a polyvinylidene difluoride membrane with semidry transfer (Bio-Rad #1704272), the membrane was blocked with 10% milk in phosphate-buffered saline with 0.1% Tween-20 (PBS-T) for 1 h at room temperature. The membrane was cut and probed with primary antibodies at a 1:1,000 dilution in 2% milk in PBS-T overnight at 4 °C. The next day, the membrane was washed thrice with PBS-T for 10 min, incubated with secondary antibody at a 1:10,000 dilution for 2 h at room temperature, and washed again thrice with PBS-T for 10 min. Chemiluminescent signal was detected with SuperSignal West Pico PLUS Chemiluminescent Substrate (Thermo Fisher Scientific #34580) using a Chemidoc XRS+ System (Bio-Rad) or an iBright Imaging System (Thermo Fisher Scientific).

Primary antibodies were used to detect the following proteins: SARS-CoV-2 Spike (Sino Biological #40591-T62), SARS-CoV-2 RdRp (Genetex #GTX135467), and GAPDH (Thermo Fisher Scientific #MA5-15738). Horseradish peroxidase (HRP)-conjugated secondary antibodies were used to detect mouse and rabbit primary antibodies (Thermo Fisher Scientific, #62-6520 and #31460, respectively).

### Enzyme-Linked Immunospot (ELISpot) Assay.

Cells were obtained from spleens by passage through a 70 μm nylon mesh cell strainer (Fisher Scientific #22-363-548) using a 3 mL syringe plunger (Fisher Scientific #14-823-435). Red blood cells were lysed with ACK lysing buffer (Thermo Fisher Scientific #A1049201) and cells were resuspended in RPMI 1640 (Corning #10-040-CV) media supplemented with 10% FBS and 1× Penicillin/Streptomycin (Complete RPMI). Lung cells were obtained by mincing the tissue with surgical scissors and digesting it with 2 to 5 mg/mL collagenase A (Sigma-Aldrich #10103586001) in complete RPMI media at 37 °C for 60 to 80 min with mixing every 10 min. The digested tissue was processed in a manner identical to spleen samples to obtain lung cells.

ELISpot was performed using a mouse IFNγ/TNFα or mouse IFNγ ELISpot kit (CTL #mIFNgTNFa-1 M/10 or #mIFNg-1 M) according to the manufacturer’s instructions. Cells were stimulated with a sliding window overlapping 15mer pool of SARS-CoV-2 Spike RBD or RdRp peptides (JPT Peptide Technologies GmbH #PM-WCPV-S-RBD-2, and #PM-WCPV-NSP12-2). Negative control wells contained unstimulated cells and positive control wells were stimulated with a cocktail of phorbol 12-myristate 13-acetate and ionomycin. Experimental wells were stimulated with 1 μg/mL Spike RBD or RdRp peptide pools in complete RPMI. Cells were incubated at 37 °C for 20 to 22 h before plates were developed. Plates were scanned and spot counts were analyzed by CTL. The number of spots in negative control wells was subtracted from experimental wells to determine the number of antigen-specific spot-forming cells.

### ICS.

For murine peripheral blood mononuclear cell (PBMC) isolation, 150 µL of whole peripheral blood was collected from mice in heparin-coated collection vials (Fisher Scientific #13-680-62) by the retro-orbital technique using heparin-coated capillary tubes. For red blood cell lysis, 100 µL of whole blood was mixed with 3 mL of ACK lysis buffer and incubated for 3 min at room temperature. ACK was quenched by adding 10 mL of fluorescence-activated cell sorting (FACS) buffer (5% FBS in PBS without Ca/Mg). Cell suspensions were centrifuged at 450 ×*g* for 4 min at 4 °C and decanted. 3 cycles of ACK lysis were performed for PBMC isolation.

Splenocytes were prepared as described above. Stimulation was performed in 96-well U bottom plates in the presence of brefeldin A (Biolegend #420601) for 6 h at 37 °C. Cells were stimulated with overlapping 15mer RBD and RdRp peptide pools at 1 μg/mL (JPT Peptide Technologies GmbH #PM-WCPV-S-RBD-2, and #PM-WCPV-NSP12-2, respectively). Individual peptides for deconvolution studies were ordered from JPT, resuspended in DMSO, and tested at 1 µg/mL or as indicated. Cells were then stored at 4 °C for up to 16 h until staining. NetMHCpan4.1 was used as the MHC binding prediction tool to generate RdRp peptide libraries (32). Peptides with predicted top 0.5% ranking were selected as the strong binders for downstream functional tests.

Stimulated cells were stained with anti-TCRβ PerCP-Cy5.5 clone H57-597 (Biolegend #109228), anti-CD4 PE-Cy7 clone RM4-5 (Biolegend #100528), and anti-CD8+ PE clone 53-6.7 (Biolegend #100708) at a 1:100 dilution for 15 min on ice. Cells were washed with FACS buffer, then fixed and permeabilized with Cytofix/Cytoperm buffer (BD #554714) for 20 min on ice. Cells were washed with permeabilization buffer, then stained with anti-IFNγ APC clone XMG1.2 and anti-TNFα FITC clone MP6-XT22 (Biolegend #505810 and #506304) at a 1:50 dilution in permeabilization buffer for 30 min on ice. Cells were washed with permeabilization buffer and resuspended in FACS buffer before analysis.

### AIM Assays.

Splenocytes were prepared as described above. Stimulation was performed in 96-well U bottom plates for 16 to 24 h at 37 °C. Cells were stained with anti-TCRbeta PerCP-Cy5.5 clone H57-597, anti-CD4 PE-Cy7 clone RM4-5, anti-CD8 PE clone 53-6.7, anti-CD69 FITC clone H1.2F3, anti-CD137 (4-1BB) APC clone 17B5 (Biolegend #109228, #100528, #100708, #104506, and #106110), and anti-CD134 (OX40) Super Bright 780 clone OX-86 (Invitrogen #78-1341-82) at a 1:200 dilution for 20 min on ice. Cells were washed with FACS buffer and resuspended before analysis.

### Flow Cytometry Analysis.

0.5 × 10^6^ murine PBMC or splenocytes were stained in 100 µL of FACS buffer supplemented with 1:200 diluted fluorochrome-conjugated antibodies and 1:100 diluted Fc-block (anti-mouse CD16/32; Biolegend #101319). After a 20 min incubation at 4 °C, samples were centrifuged and washed twice with 1 mL of FACS buffer before resuspension in 500 µL of FACS buffer + 250 ng/mL DAPI before data acquisition. For mouse T cell phenotyping, cells were stained with anti-CD3 FITC clone 145-2C11 (Biolegend #100203), anti-CD8+ PE-Cy7 clone 53-6.7 (Biolegend #100721), anti-CD4 APC clone GK1.5 (Biolegend #100412), anti-CD44 AlexaFluor700 clone IM7 (Biolegend #103025), anti-CD62L BV605 clone MEL-14 (Biolegend #104437), anti-CD127 APC-Cy7 clone A7R34 (Biolegend #135039), anti-KLRG1 PE clone 2F1 (Biolegend #138407). For flow cytometry analysis of KP cell surface MHC I levels, cells were trypsinized and 0.5 × 10^6^ cells were stained with anti-mouse MHC I FITC clone M1/42 (Biolegend #125507) at a 1:200 dilution in FACS buffer for 20 min at 4 °C. KP cells were washed with FACS buffer and resuspended in 500 µL of FACS buffer + 250 ng/mL DAPI before data acquisition. Flow cytometry data were acquired on a five-laser BD LSRII or BD Canto and analyzed using FlowJo software. Cells were gated for viable (DAPI-) singlets for analysis.

### Spike S1 Enzyme-Linked Immunosorbent Assay (ELISA).

ELISA plates (Corning #07-200-721) were prepared by coating each well with 50 μL recombinant SARS-CoV-2 Spike S1 (Sino Biological #40591-V08H) at a 1 μg/mL concentration in carbonate-bicarbonate buffer (Sigma-Aldrich #C3041-50CAP) overnight at 4 °C. Coated 96-well plates were blocked with PBS containing 1% BSA (%w/v; Fisher Scientific #BP9703100), and 0.05% Tween-20 (%v/v; Fisher Scientific #BP337-500) at room temperature for 1 h or overnight at 4 °C. All serum samples and secondary antibodies were diluted in assay buffer consisting of PBS with 0.1% BSA and 0.025% Tween-20. Coated plates were washed with DPBS containing 0.1% Tween-20 (%v/v; PBS-T) twice for 3 min. Plates were then washed twice quickly with PBS-T before the addition of 50 μL serially diluted serum. 6 to 8 wells per plate were incubated with assay buffer containing no primary antibody as a background control. Plates were incubated for 1 to 2 h at room temperature on an orbital shaker. Plates were washed with PBS-T twice for 3 min, then twice quickly before the addition of 50 μL 1:4,000 goat anti-mouse HRP secondary antibody (Thermo Fisher Scientific #62-6520). Secondary antibody was incubated for 1 h at room temperature with shaking. Plates were then washed once with PBS-T for 3 min, then four times quickly. After a final PBS wash (no Tween-20), 100 μL of 1-Step Ultra TMB ELISA Substrate (Thermo Fisher Scientific #34028) was added to each well. Plates were covered to protect them from light and incubated at room temperature for 30 min with shaking. Signal development was stopped by the addition of 100 μL 1 M sulfuric acid (Sigma-Aldrich #1603131000) and the optical density at 450 nm (OD450) was measured with a ClarioStar plate reader (BMG Labtech).

### Transduction of KP4662 Target Cells.

The truncated RdRp coding sequence used in mRNA vaccine construct was codon optimized, ordered as a gBlock (IDT) and cloned into an pCCL lentiviral construct under an MNDU3 promoter sequence followed by an IRES-mStrawberry selectable marker. KP4662 cells were transduced via lentivirus and single-cell sorted by FACS. Two candidate clones were selected following expansion and evaluation of RdRp protein expression by immunoblot analysis.

### Generation of TCR-Engineered Murine T Cells.

TCR alpha and beta chain sequences from high frequency, activated cells returned from single-cell sequencing or the pmel-1 TCR (33) were ordered as gBlocks (IDT or Twist) and cloned into an MSCV backbone vector. Retrovirus was prepared with an ecotrophic coat and was harvested from transfected 293 T cells.

T cells were enriched from C57BL/6 splenocytes using CD3+ magnetic selection beads (EasySep Mouse T Cell Isolation Kit, Stemcell Technologies #19851A) and plated in RPMI 1640 media supplemented with 10% FBS, 1X GlutaMAX, 55 µM β-mercaptoethanol, 50 U/mL of human IL-2 (PeproTech #200-02), and 1 ng/mL murine IL-7 (Peprotech #217-17) overnight with CD3/CD28 murine T cell activation/expansion Dynabeads (Thermo Fisher #11452D). 24 h later, murine T cells were transduced via retrovirus encoding candidate TCRs. Viruses were thawed at 37 °C. Media were removed from PBMCs and replaced with unconcentrated retroviral supernatant and 5 µg/mL of polybrene (Sigma #H9268). Splenocytes were centrifuged at 1,455 ×*g* at 30 °C for 90 min. After centrifugation, retroviral supernatant was replaced with media and incubated at 37 °C overnight. Spinfection was repeated the following day. 24 h after the second spinfection, the cell and beads were replated with fresh media. 48 h after washing, beads were removed by magnetic separation (BD #552811) and replated at 1.5 × 10^6^ cells/mL. Cells were expanded for one wk with intermittent half media changes before use in functional assays.

### T Cell Cocultures.

TCR-engineered murine CD3+ T cells were cocultured with B16F10 cells or KP4662 expressing truncated RdRp at an 8:1 effector to target cell ratio. B16F10 murine melanoma cells were used as antigen-presenting cells in cocultures testing TCR-engineered T cells where RdRp peptides or peptide pools were added exogenously for IFNγ ELISA analysis. For KP4662 cocultures, KP4662 were pretreated with 100 U/mL murine IFNβ (PBL #12405) to enhance MHC I cell surface levels. All cocultures were performed in target cell media without added cytokines. Cocultures were incubated at 37 °C for 48 h before IFNγ ELISA analysis or 72 h before crystal violet staining cell survival analysis.

### Crystal Violet Staining.

Following T cell coculture with KP4662 (KP) target cells, media from 96-well plates was decanted and adherent target cells were fixed by incubation with 100% MeOH for 20 min at −20 °C. Plates were washed three times with PBS and stained with a 0.05% w/v solution of Crystal Violet (Fisher Scientific #C581-25) prepared in dH_2_O. Staining was performed by incubating plates on a rocker at room temperature for 30 min. Plates were washed with dH_2_O before analysis.

### IFNγ ELISA.

Supernatant was harvested from cell cultures 48 h after peptide stimulation or the initiation of the coculture. Supernatant was stored at −70 °C before analysis. Supernatant IFNγ concentration was quantified via sandwich cytokine ELISA using the OptEIA Reagent Set B (BD #550534) and OptEIA IFNγ (AN-18) ELISA kit (BD #551866).

### Single-cell RNA Sequencing.

#### Sample preparation.

Freshly isolated spleens from C57BL/6 mice (n = 5) were mashed through a 70 µm nylon mesh strainer using a 1 mL syringe plunger to obtain a single-cell suspension. For red blood cell lysis, cell suspensions were centrifuged at 300 ×*g* for 2 min at 4 °C and decanted. Cell pellets were resuspended in 3 mL of ACK lysis buffer and incubated for 3 min at room temperature. ACK was quenched by adding 10 mL of RPMI with 10% FBS. Cells were centrifuged and resuspended in scRNAseq buffer (PBS without Ca/Mg + 0.04% BSA). CD8+ cells were isolated by negative selection using a mouse CD8a+ T Cell Isolation Kit (Miltenyi #130-104-075) and immuno-magnetic separation (OctoMACS; Miltenyi #130042108). Enriched CD8+ cells were centrifuged and washed twice with scRNAseq buffer. CD8+ cell-enriched splenocytes from 5 mice were pooled 1:1:1:1:1 at 1,000 cells/µL in 1 mL of scRNAseq buffer.

#### Library construction and NGS.

Single-cell capture and library construction was performed at the UCLA Technology Center for Genomics and Bioinformatics core facility. Samples were counted using a Countess II Automated Cell Counter (Thermo Fisher Scientific) and hemocytometer for cell concentration using Trypan Blue stain 0.4% (Invitrogen). The cell sample was confirmed to contain >70% viable cells. Single-cell gene expression libraries (GEX) were created using Chromium Next GEM Single Cell 5' (v2 Chemistry; 10× Genomics #PN-1000263), the TCR V(D)J amplification was performed using the 10× Chromium Single Cell mouse TCR amplification kit (10x Genomics #PN-1000254), Chromium Next GEM Chip G Single Cell Kit (10× Genomics #PN-1000127), and Chromium i7 Multiplex Kit (10× Genomics #PN-120262) according to the manufacturer’s instructions. Briefly, cells were loaded to target 10,000 cells to form GEMs and barcode individual cells. GEMs were then cleaned, and cDNA and libraries were also created according to the manufacturer’s instructions. Library quality was assessed using 4,200 TapeStation System with both D1000 and D5000 ScreenTapes (Agilent) and Qubit 2.0 (Invitrogen) for size distribution and concentration. Samples were sequenced using Paired End sequencing (91+19+24+91) Novaseq 6,000 (Illumina). Fastq.gz files were trimmed and generated to 10× Genomics specifications using bcl2fastq (Illumina). 200 M reads (GEX) and 50 M reads (TCR V(D)J) were targeted for each sample, targeting 20,000 reads per cell.

#### Bioinformatics analysis.

For single-cell gene expression analysis, samples were aligned to the mm10 mouse genome using CellRanger (version 4.0.0). For V(D)J library TCR analysis, the artifact sequence "CAGATCTCGGTGGTCGCCGTATCAT" was detected and removed from all R2 reads. Once artifact sequences were removed, samples were aligned to the mouse Cell Ranger V(D)J compatible reference (refdata-cellranger-vdj-GRCm38-alts-ensembl-5.0.0) using CellRanger (version 6.1.2). The aligned datasets were processed with the Seurat (version 4.0.4) R package in R Studio (version 2021.09.1, R version 4.1.2). For quality control, cells with greater than 20% mitochondrial gene expression and cells with a number of unique molecular identifiers (nUMI) lower than 200 were excluded. Features (genes) not supported by a minimum of 20 cells were excluded. Principal component analysis dimensionality reduction was applied. Subsequently, based on the top 30 principal components, we generated the UMAP-based visualization, nearest-neighbor computation, and cell clustering.

#### Quantification and statistical analysis.

Data are presented as mean ± SD with number of biological replicates indicated in figure legends. Comparisons of two groups were evaluated using the unpaired two-tailed unpaired *t* test and *P* values less than 0.05 were considered significant. Comparisons of more than two groups were evaluated using one-way ANOVA followed by Bonferroni’s multiple comparison test and *P* values less than 0.05/m, where m is the total number of possible comparisons, were considered significant.

## Supplementary Material

Appendix 01 (PDF)

## Data Availability

All unique/stable reagents generated in this study are available from the Lead Contacts with a completed materials transfer agreement. Single-cell RNA sequencing and TCR sequencing data for RdRp vaccine responses are available at the NCBI Gene Expression Omnibus (GSE248086) (31). All other data are included in the manuscript and/or SI Appendix.

## References

[1] D. Laczkó , A single immunization with nucleoside-modified mRNA vaccines elicits strong cellular and humoral immune responses against SARS-CoV-2 in mice. Immunity **53**, 724–732.e7 (2020). 32783919 10.1016/j.immuni.2020.07.019 PMC7392193

[2] R. R. Goel , mRNA vaccines induce durable immune memory to SARS-CoV-2 and variants of concern. Science **374**, abm0829 (2021). 34648302 10.1126/science.abm0829 PMC9284784

[3] A. Muik , Progressive loss of conserved spike protein neutralizing antibody sites in Omicron sublineages is balanced by preserved T cell immunity. Cell Rep. **42**, 112888 (2023). 37527039 10.1016/j.celrep.2023.112888

[4] K. Lederer , SARS-CoV-2 mRNA vaccines foster potent antigen-specific germinal center responses associated with neutralizing antibody generation. Immunity **53**, 1281–1295.e5 (2020). 33296685 10.1016/j.immuni.2020.11.009 PMC7680029

[5] K. McMahan , Correlates of protection against SARS-CoV-2 in rhesus macaques. Nature **590**, 630–634 (2021). 33276369 10.1038/s41586-020-03041-6 PMC7906955

[6] E. M. Bange , CD8+ T cells contribute to survival in patients with COVID-19 and hematologic cancer. Nat. Med. **27**, 1280–1289 (2021). 34017137 10.1038/s41591-021-01386-7 PMC8291091

[7] P. Moss, The T cell immune response against SARS-CoV-2. Nat. Immunol. **23**, 186–193 (2022). 35105982 10.1038/s41590-021-01122-w

[8] H. Qi, B. Liu, X. Wang, L. Zhang, The humoral response and antibodies against SARS-CoV-2 infection. Nat. Immunol. **23**, 1008–1020 (2022). 35761083 10.1038/s41590-022-01248-5

[9] R. Keeton , T cell responses to SARS-CoV-2 spike cross-recognize Omicron. Nature **603**, 488–492 (2022). 35102311 10.1038/s41586-022-04460-3 PMC8930768

[10] C. M. Arieta , The T-cell-directed vaccine BNT162b4 encoding conserved non-spike antigens protects animals from severe SARS-CoV-2 infection. Cell **186**, 2392–2409.e21 (2023). 37164012 10.1016/j.cell.2023.04.007 PMC10099181

[11] P. A. Nesterenko , HLA-A∗02:01 restricted T cell receptors against the highly conserved SARS-CoV-2 polymerase cross-react with human coronaviruses. Cell Rep. **37**, 110167 (2021). 34919800 10.1016/j.celrep.2021.110167 PMC8660260

[12] A. Grifoni , Targets of T cell responses to SARS-CoV-2 coronavirus in humans with COVID-19 disease and unexposed individuals. Cell **181**, 1489–1501.e15 (2020). 32473127 10.1016/j.cell.2020.05.015 PMC7237901

[13] A. Tarke , Comprehensive analysis of T cell immunodominance and immunoprevalence of SARS-CoV-2 epitopes in COVID-19 cases. Cell Rep. Med. **2**, 100204 (2021). 33521695 10.1016/j.xcrm.2021.100204 PMC7837622

[14] F. P. Polack , Safety and efficacy of the BNT162b2 mRNA covid-19 vaccine. N. Engl. J. Med. **383**, 2603–2615 (2020). 33301246 10.1056/NEJMoa2034577 PMC7745181

[15] L. R. Baden , Efficacy and safety of the mRNA-1273 SARS-CoV-2 vaccine. N. Engl. J. Med. **384**, 403–416 (2021). 33378609 10.1056/NEJMoa2035389 PMC7787219

[16] C. P. Arevalo , A multivalent nucleoside-modified mRNA vaccine against all known influenza virus subtypes. Science **378**, 899–904 (2022). 36423275 10.1126/science.abm0271 PMC10790309

[17] N. Pardi, M. J. Hogan, F. W. Porter, D. Weissman, mRNA vaccines - a new era in vaccinology. Nat. Rev. Drug Discov. **17**, 261–279 (2018). 29326426 10.1038/nrd.2017.243 PMC5906799

[18] S. Tahtinen , IL-1 and IL-1ra are key regulators of the inflammatory response to RNA vaccines. Nat. Immunol. **23**, 532–542 (2022). 35332327 10.1038/s41590-022-01160-y

[19] M. G. Alameh , Lipid nanoparticles enhance the efficacy of mRNA and protein subunit vaccines by inducing robust T follicular helper cell and humoral responses. Immunity **54**, 2877–2892.**e7** (2021). 34852217 10.1016/j.immuni.2021.11.001 PMC8566475

[20] R. Verbeke, M. J. Hogan, K. Loré, N. Pardi, Innate immune mechanisms of mRNA vaccines. Immunity **55**, 1993–2005 (2022). 36351374 10.1016/j.immuni.2022.10.014 PMC9641982

[21] C. Li , Mechanisms of innate and adaptive immunity to the Pfizer-BioNTech BNT162b2 vaccine. Nat. Immunol. **23**, 543–555 (2022). 35288714 10.1038/s41590-022-01163-9 PMC8989677

[22] J. Mateus , Low-dose mRNA-1273 COVID-19 vaccine generates durable memory enhanced by cross-reactive T cells. Science **374**, eabj9853 (2021). 34519540 10.1126/science.abj9853 PMC8542617

[23] S. Kreiter , Increased antigen presentation efficiency by coupling antigens to MHC class I trafficking signals. J. Immunol. **180**, 309–318 (2008). 18097032 10.4049/jimmunol.180.1.309

[24] N. Pardi, H. Muramatsu, D. Weissman, K. Karikó, In vitro transcription of long RNA containing modified nucleosides. Methods Mol. Biol. **969**, 29–42 (2013). 23296925 10.1007/978-1-62703-260-5_2

[25] M. A. Maier , Biodegradable lipids enabling rapidly eliminated lipid nanoparticles for systemic delivery of RNAi therapeutics. Mol. Ther. **21**, 1570–1578 (2013). 23799535 10.1038/mt.2013.124 PMC3734658

[26] S. Ndeupen , The mRNA-LNP platform’s lipid nanoparticle component used in preclinical vaccine studies is highly inflammatory. iScience **24**, 103479 (2021). 34841223 10.1016/j.isci.2021.103479 PMC8604799

[27] A. Lemieux , Enhanced detection of antigen-specific T cells by a multiplexed AIM assay. Cell Rep. Methods **4**, 100690 (2024). 38228152 10.1016/j.crmeth.2023.100690 PMC10831934

[28] A. X. Le , Cytotoxic T cell responses in HLA-A2.1 transgenic mice. Recognition of HLA alloantigens and utilization of HLA-A2.1 as a restriction element. J. Immunol. **142**, 1366–1371 (1989). 2464645

[29] E. E. Walsh , Safety and immunogenicity of two RNA-based Covid-19 vaccine candidates. N. Engl. J. Med. **383**, 2439–2450 (2020). 33053279 10.1056/NEJMoa2027906 PMC7583697

[30] A. B. Vogel , BNT162b vaccines protect rhesus macaques from SARS-CoV-2. Nature **592**, 283–289 (2021). 33524990 10.1038/s41586-021-03275-y

[31] E. Abt, W. Tran, T. T. Wu, Data from “Gene expression profile at the single cell level of CD8+ cells isolated from mice immunized with a mRNA vaccine encoding SARS-CoV-2 RdRp”. NCBI Gene Expression Omnibus. https://www.ncbi.nlm.nih.gov/geo/query/acc.cgi?acc=GSE248086. Deposited 17 November 2023.

[32] B. Reynisson, B. Alvarez, S. Paul, B. Peters, M. Nielsen, NetMHCpan-4.1 and NetMHCIIpan-4.0: Improved predictions of MHC antigen presentation by concurrent motif deconvolution and integration of MS MHC eluted ligand data. Nucleic Acids Res. **48**, W449–W454 (2020). 32406916 10.1093/nar/gkaa379 PMC7319546

[33] J. D. Abad , T-cell receptor gene therapy of established tumors in a murine melanoma model. J. Immunother. **31**, 1–6 (2008). 18157006 10.1097/CJI.0b013e31815c193f PMC2235937

[34] Z. Zhuang , Mapping and role of T cell response in SARS-CoV-2-infected mice. J Exp Med **218**, e20202187 (2021). 33464307 10.1084/jem.20202187 PMC7814348

[35] L. A. Farrington, T. A. Smith, F. Grey, A. B. Hill, C. M. Snyder, Competition for antigen at the level of the APC is a major determinant of immunodominance during memory inflation in murine cytomegalovirus infection. J. Immunol. **190**, 3410–3416 (2013). 23455500 10.4049/jimmunol.1203151 PMC3608834

[36] Z. Yan , Next-generation IEDB tools: A platform for epitope prediction and analysis. Nucleic Acids Res. **52**, W526–W532 (2024). 38783079 10.1093/nar/gkae407 PMC11223806

[37] N. Pardi , Expression kinetics of nucleoside-modified mRNA delivered in lipid nanoparticles to mice by various routes. J. Control Release **217**, 345–351 (2015). 26264835 10.1016/j.jconrel.2015.08.007 PMC4624045

[38] W. Dong , The K18-Human ACE2 transgenic mouse model recapitulates non-severe and severe COVID-19 in response to an infectious dose of the SARS-CoV-2 virus. J. Virol. **96**, e0096421 (2022). 34668775 10.1128/JVI.00964-21 PMC8754221

[39] I. N. Pardieck , A third vaccination with a single T cell epitope confers protection in a murine model of SARS-CoV-2 infection. Nat. Commun. **13**, 3966 (2022). 35803932 10.1038/s41467-022-31721-6 PMC9267705

[40] H. Watanabe, K. Numata, T. Ito, K. Takagi, A. Matsukawa, Innate immune response in Th1- and Th2-dominant mouse strains. Shock **22**, 460–470 (2004). 15489639 10.1097/01.shk.0000142249.08135.e9

[41] U. Sahin , Personalized RNA mutanome vaccines mobilize poly-specific therapeutic immunity against cancer. Nature **547**, 222–226 (2017). 28678784 10.1038/nature23003

[42] E. S. Winkler , SARS-CoV-2 infection of human ACE2-transgenic mice causes severe lung inflammation and impaired function. Nat. Immunol. **21**, 1327–1335 (2020). 32839612 10.1038/s41590-020-0778-2 PMC7578095

[43] F. S. Oladunni , Lethality of SARS-CoV-2 infection in K18 human angiotensin-converting enzyme 2 transgenic mice. Nat. Commun. **11**, 6122 (2020). 33257679 10.1038/s41467-020-19891-7 PMC7705712

[44] C. K. Yinda , K18-hACE2 mice develop respiratory disease resembling severe COVID-19. PLoS Pathog. **17**, e1009195 (2021). 33465158 10.1371/journal.ppat.1009195 PMC7875348

[45] E. S. Winkler , SARS-CoV-2 causes lung infection without severe disease in human ACE2 knock-in mice. J. Virol. **96**, e0151121 (2022). 34668780 10.1128/JVI.01511-21 PMC8754206

[46] S. Weingarten-Gabbay , Profiling SARS-CoV-2 HLA-I peptidome reveals T cell epitopes from out-of-frame ORFs. Cell **184**, 3962–3980.e17 (2021). 34171305 10.1016/j.cell.2021.05.046 PMC8173604

[47] E. Vidor, The nature and consequences of intra- and inter-vaccine interference. J. Comp. Pathol. **137**, S62–S66 (2007). 17560593 10.1016/j.jcpa.2007.04.014

[48] S. Tahtinen , IL-1 and IL-1ra are key regulators of the inflammatory response to RNA vaccines. Nat. Immunol. **23**, 532–542 (2022). 35332327 10.1038/s41590-022-01160-y

[49] M. Baiersdörfer , A facile method for the removal of dsRNA contaminant from in vitro-transcribed mRNA. Mol. Ther. Nucleic Acids **15**, 26–35 (2019). 30933724 10.1016/j.omtn.2019.02.018 PMC6444222

[50] B. Fallet , Interferon-driven deletion of antiviral B cells at the onset of chronic infection. Sci. Immunol. **1**, eaah6817 (2016). 27872905 10.1126/sciimmunol.aah6817 PMC5115616

[51] E. A. Moseman, T. Wu, J. C. de la Torre, P. L. Schwartzberg, D. B. McGavern, Type I interferon suppresses virus-specific B cell responses by modulating CD8+ T cell differentiation Sci. Immunol. **1**, eaah3565 (2016). 27812556 10.1126/sciimmunol.aah3565 PMC5089817

[52] S. Sammicheli , Inflammatory monocytes hinder antiviral B cell responses. Sci. Immunol. **1**, eaah6789 (2016). 27868108 10.1126/sciimmunol.aah6789 PMC5111729

[53] N. Palacio , Early type I IFN blockade improves the efficacy of viral vaccines. J. Exp. Med. **217**, e20191220 (2020). 32820330 10.1084/jem.20191220 PMC7953731

[54] C. Muñoz-Fontela , Advances and gaps in SARS-CoV-2 infection models. PLoS Pathog. **18**, e1010161 (2022). 35025969 10.1371/journal.ppat.1010161 PMC8757994

