# Supplementary material for: Staggered immunization with mRNA vaccines encoding SARS-CoV-2 polymerase or spike antigens broadens the T cell epitope repertoire
Source: Proc Natl Acad Sci U S A. 2024 Nov 26;121(49):e2406332121. doi: 10.1073/pnas.2406332121 (PMC11626164; doi:10.1073/pnas.2406332121)
Supplement: Supplementary file 1 — Appendix 01 (PDF) [file pnas.2406332121.sapp.pdf]

Figure S1

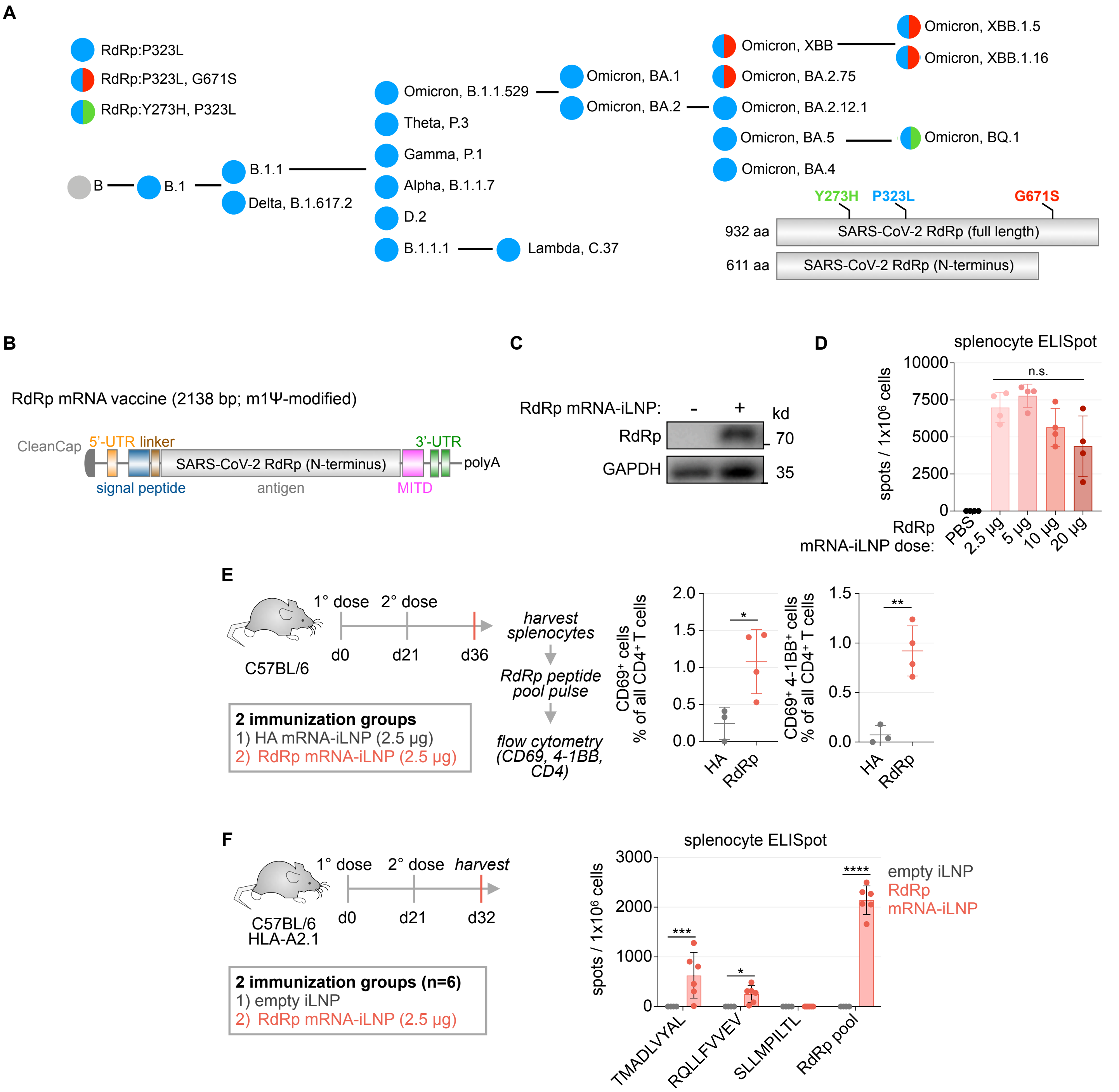

Figure S1 | RdRp-specific T cell responses elicited by a RdRp mRNA-iLNP vaccine. Related to Figure 1. (**A**) Schematic of non-synonymous mutations in the SARS-CoV-2 RdRp (NSP12) protein across variants of concern. Variant mutations were retrieved from BV-BRC (https://www.bv-brc.org/view/VariantLineage/). (**B**) RdRp-encoding mRNA vaccine construct design. (**C**) Immunoblot analysis of HEK293T cells transfected with RdRp mRNA-iLNP. (**D**) IFNγ ELISpot analysis of day 28 splenocytes pulsed with a RdRp peptide pool (unpaired t test; mean±SD; n=6; unpaired t test). (**E**) Activation-induced marker (AIM) assay for RdRp-specific $CD4^+$ T cells from splenocytes isolated from mice immunized with 2.5 µg RdRp or HA mRNA iLNP (mean±SD; n=3-4; unpaired t test). (**F**) Experiment to test the T cell immunogenicity of the RdRp mRNA-iLNP in HLA-A2.1 transgenic mice. IFNγ ELISpot analysis of day 32 splenocytes pulsed with RdRp peptides or a peptide pool (unpaired t test; mean±SD; n=6; unpaired t test). n.s.: not significant; * P<0.05; ** P<0.01; *** P<0.001; **** P<0.0001.

Figure S2

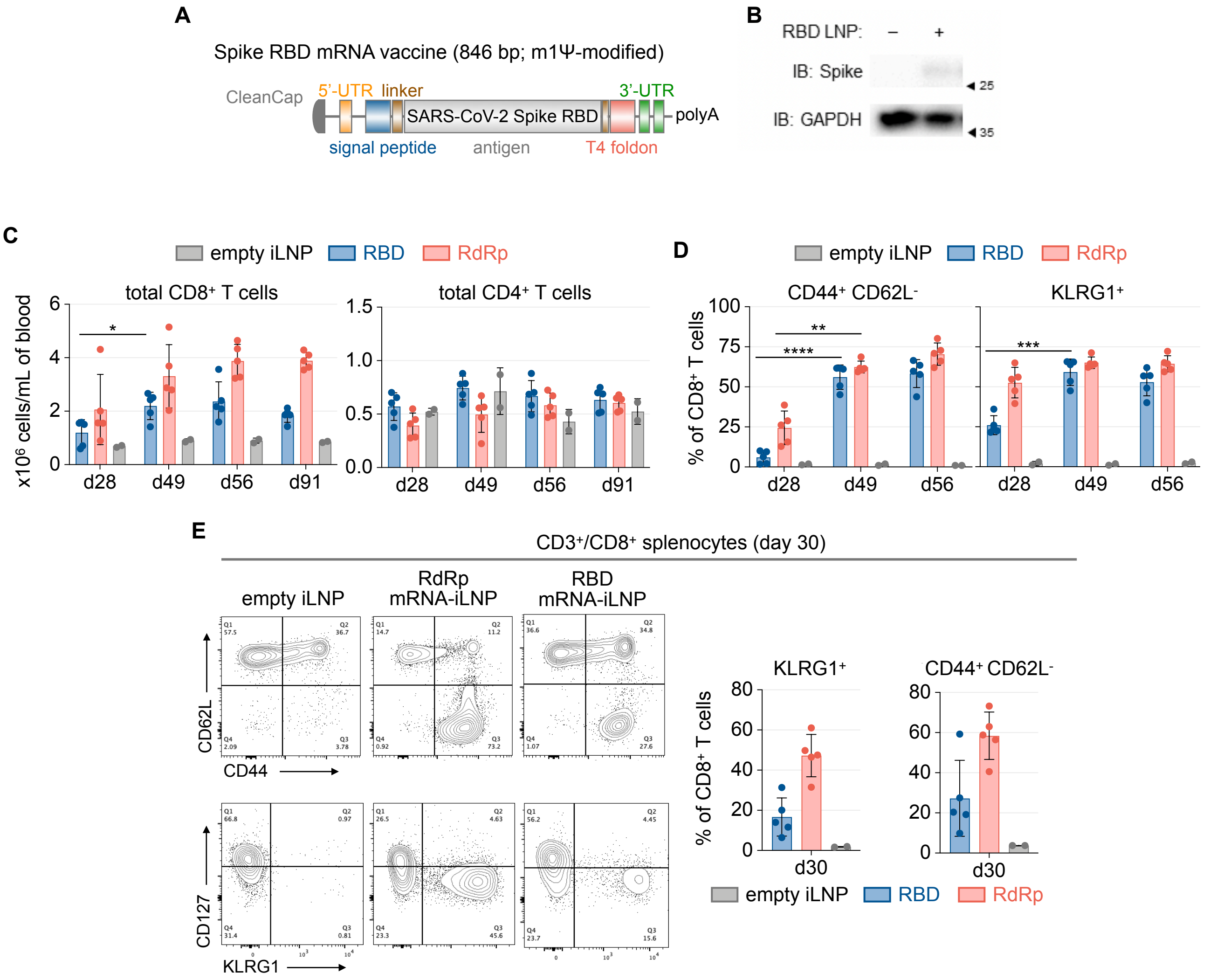

Figure S2 | T cell phenotype following RBD or RdRp mRNA vaccine immunization. Related to Figure 1. (**A**) SARS-CoV-2 Spike RBD-encoding mRNA-iLNP design. (**B**) Immunoblot analysis of HEK293T cells transfected with Spike RBD mRNA-iLNP. (**C**) Total $CD8^+$ or $CD4^+$ T cells in peripheral blood following immunization as in **Figure 1D** (mean±SD; unpaired t test). (**D**) Peripheral blood $CD3^+$/$CD8^+$ T cell phenotype following immunization as in **Figure 1D** (mean±SD; one way ANOVA). (**E**) Splenocyte $CD3^+$/$CD8^+$ T cell phenotype on day 30 (mean±SD). * $P<0.05$; ** $P<0.01$; *** $P<0.001$; **** $P<0.0001$.

Figure S3

A

	1	2	3	4	5	6
7	NQDVNLHSSRL	SVAALTNNVAF	FSNYQHEETI	YSLLMPILTL	VAALTNNVAF	SMSYEDQDAL
8	YSDVENGGSL	SNYQHEETI	AGIVGVLTL	AALTNNVAF	SSVELKHFF	AAISDYDYY
9	RQLLFVVEV	MSYEDQDAL	FAYTKRNVI	SNYQHEETI	VVDSYYSLL	**STGYHFREL**
10	SSRLSFKEL	TNRQFHQKL	VVYRAFDI	LVYALRHF	ILRVYANL	VVDSYYSL
11	FNVLFSTV	TVFPPTSF	**TGYHFREL**	VNLHSSRL	FAYTKRNV	VIPTITQM

B

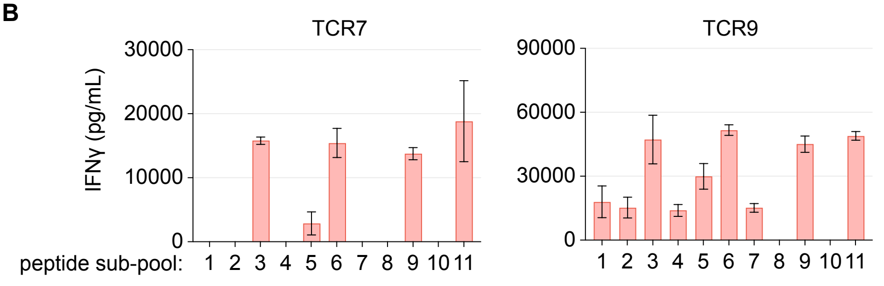

C

V(D)J clonotypes enriched in ≥ 5 cells (**Figure 2E**)	29
TCRs cloned and transduced in $CD3^+$ splenocytes	14
TCRs with confirmed RdRp peptide pool reactivity	10

D

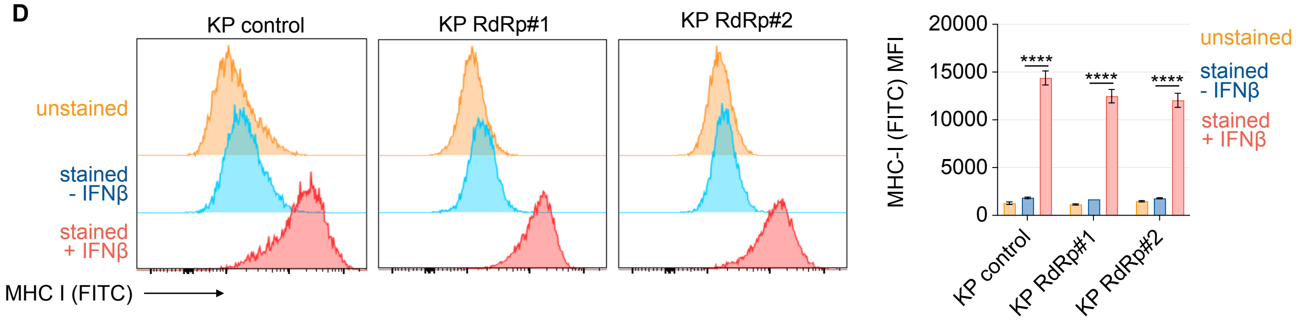

E

allele	peptide length	core sequence	peptide sequence	netMHCpan_el score	netMHCpan_el percentile
$H2\text{-}IA^b$	15	VENPDILRV	YDFVENPDILRVYAN	0.4155	2.4
$H2\text{-}IA^b$	15	MHAASGNLL	DPAMHAASGNLLLD	0.405	2.5
$H2\text{-}IA^b$	15	FVDGVPFVV	RKIFVDGVPFVVSTG	0.373	2.7
$H2\text{-}IA^b$	15	FIQTTPGSG	FGDFIQTTPGSGVPV	0.3565	2.9
$H2\text{-}IA^b$	15	LKYAISAKN	QMNLKYAISAKNRAR	0.3466	3
$H2\text{-}IA^b$	15	VENPDILRV	WYDFVENPDILRVYA	0.3357	3.1
$H2\text{-}IA^b$	15	FIQTTPGSG	DFGDFIQTTPGSGVP	0.3409	3.1
$H2\text{-}IA^b$	15	MHAASGNLL	ADPAMHAASGNLLL	0.3149	3.4
$H2\text{-}IA^b$	15	FVDGVPFVV	VRKIFVDGVPFVVST	0.315	3.4
$H2\text{-}IA^b$	15	LKYAISAKN	TQMNLKYAISAKNRA	0.2788	4
$H2\text{-}IA^b$	15	YAADPAMHA	ELLVYAADPAMHAAS	0.2745	4.1
$H2\text{-}IA^b$	15	YAADPAMHA	LLVYAADPAMHAASG	0.2636	4.2
$H2\text{-}IA^b$	15	YRYNLPTMC	YDYYRYNLPTMCDIR	0.2443	4.6
$H2\text{-}IA^b$	15	FVDGVPFVV	LVRKIFVDGVPFVVS	0.24	4.7
$H2\text{-}IA^b$	15	VENPDILRV	DWYDFVENPDILRVY	0.2157	5.1
$H2\text{-}IA^b$	15	VYAADPAMH	KELLVYAADPAMHAA	0.2179	5.1
$H2\text{-}IA^b$	15	VENPDILRV	DFVENPDILRVYANL	0.2185	5.1
$H2\text{-}IA^b$	15	FQTVKPGNF	NVAFQTVKPGNFNK	0.2201	5.1
$H2\text{-}IA^b$	15	SFGPLVRKI	PPTSFGPLVRKIFVD	0.2136	5.2
$H2\text{-}IA^b$	15	FIQTTPGSG	GDFIQTTPGSGVPVV	0.2082	5.3

Figure S3 | De-convolution of RdRp-specific mTCRαβ congnate peptides. Related to Figure 3. (**A**) Composition of the 11 RdRp peptide sub-pools. Peptides were prioritized for testing based on predicted binding to $H2\text{-}K^b$ or $H2\text{-}D^b$ by NetMHCpan4.1. (**B**) IFNγ ELISA analysis of mTCRαβ-transduced T cells pulsed with RdRp peptide sub-pools (mean±SD; n=2). (**C**) Summary of experiments for the deconvolution of RdRp-specific TCRαβ cognate peptides. (**D**) Flow cytometry analysis of cell surface MHC I levels in KP isogenic cells treated ± 100 U/mL IFNβ for 72 h (mean±SD; n=2; unpaired t test). **** P<0.0001. (**E**) RdRp epitopes predicted to be presented by the C57BL/6 MHC II allele within the RdRp sequence encoded by the mRNA vaccine predicted by NetMHCpan4.1.

Figure S4

A–D

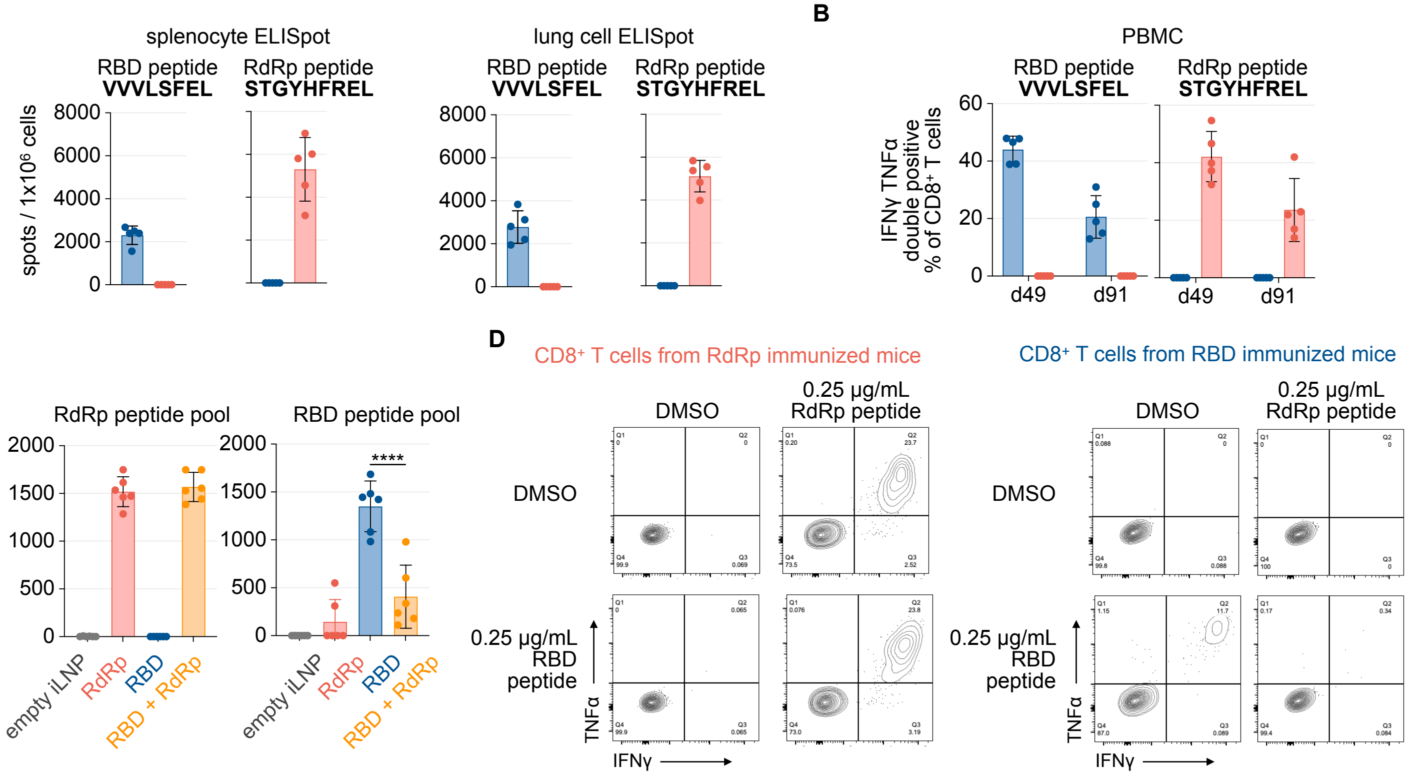

E

peptide	length	allele	netMHCpan_el score	netMHCpan_el percentile	netMHCpan_ba IC50 (nM)	netMHCpan_ba percentile	netMHCpan_ba Median rank percentile
STGYHFREL (RdRp)	9	H2-K^{b}	0.48140	0.13	146.30	0.22	0.18
STGYHFREL (RdRp)	9	H2-D^{b}	0.0375	1.9	16460.46	5.6	3.75
TGYHFREL (RdRp)	8	H2-K^{b}	0.9668	0.01	6.87	0.02	0.02
TGYHFREL (RdRp)	8	H2-D^{b}	0.0123	4.4	20466.42	8.3	6.35
VVVLSFEL (RBD)	8	H2-K^{b}	0.3886	0.2	174.67	0.24	0.22
VVVLSFEL (RBD)	8	H2-D^{b}	0.0043	8.8	25185.15	15.0	11.9

F

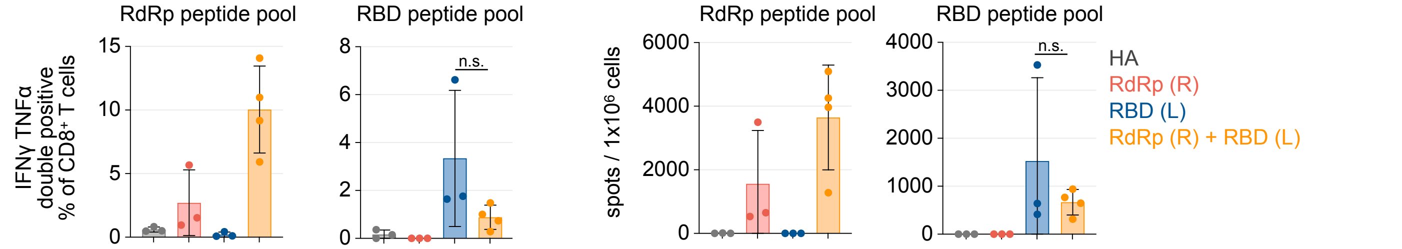

Figure S4 | RdRp and RBD mRNA vaccines individually elicit antigen-specific CD8$^+$ T cell responses against conserved epitopes in mice. Related to Figures 4 and 5. (**A**) ELISpot analysis of day 30 splenocytes and lung cells from mice immunized with RBD or RdRp mRNA-iLNP as in **Figure 1D** and restimulated *ex vivo* with individual RBD or RdRp peptides (mean±SD). (**B**) ICS analysis of CD3$^+$/CD8$^+$ PBMC from mice immunized with RBD or RdRp mRNA-iLNP isolated on experiment day 49 or 91 as in **Figure 1D** restimulated *ex vivo* with RBD or RdRp peptides (mean±SD). (**C**) ELISpot analysis of day 27 splenocytes from mice immunized with RBD and RdRp mRNA-iLNP individually or simultaneously, restimulated with RdRp or RBD peptide pools from experiment in **Figure 4A** (mean±SD; n=5; one way ANOVA). (**D**) ICS analysis of PBMC from immunized mice restimulated with RdRp (STGYHFREL) or RBD (VVVLSFEL) peptides as indicated from experiment in **Figure 4C**. (**E**) Presentation likelihood of the immunodominant epitopes encoded by each vaccine evaluated *in silico* using the NetMHCpan4.1-BA and NetMHCpan4.1-EL algorithms within the Next-Generation Immune Epitope Database and Tools (IEDB) class I T cell prediction analysis resource. (**F**) ICS and ELISpot analysis of day 32 splenocytes restimulated with RdRp or RBD peptide pools. Splenocytes were obtained from experiment in **Figure 5B**. C57BL/6 mice were immunized with HA, RBD or RdRp mRNA-iLNP individually at left (L) or right (R) gastrocnemius muscle injection sites (2.5 μg; i.m.; one way anova).

Figure S5

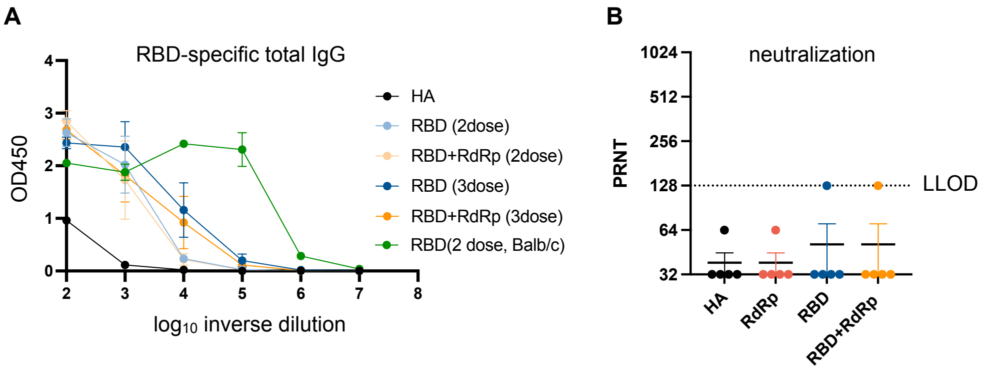

Figure S5 | SARS-CoV-2 specific antibody responses elicited by mRNA vaccine immunization. Related to Figure 6. (**A**) ELISA Spike protein specific IgG responses in sera from K18-ACE2 mice immunized with either 2 or 3 mRNA-iLNP doses. Sera from Balb/c mice receiving two Spike RBD vaccine doses were analyzed as a control. (**B**) SARS-CoV-2 neutralizing antibody titers in sera from K18-ACE2 mice receiving 3 mRNA vaccine doses as in **Figure 6D**.
